# Key Findings from Mental Health Research During the Menopause Transition for Racially and Ethnically Minoritized Women Living in the United States: A Scoping Review

**DOI:** 10.1089/jwh.2023.0276

**Published:** 2024-02-13

**Authors:** Tamara Lewis Johnson, Laura M. Rowland, Mahela S. Ashraf, Crystal T. Clark, Vonetta M. Dotson, Alicia A. Livinski, Melissa Simon

**Affiliations:** ^1^ Women's Mental Health Research Program, Office of Disparities Research and Workforce Diversity, National Institute of Mental Health, NIH, Rockville, Maryland, USA.; ^2^ Neuroscience of Mental Disorders and Aging Program, Division of Translational Research, National Institute of Mental Health, NIH, Rockville, Maryland, USA.; ^3^ Department of Psychiatry, Michigan Medicine, Ann Arbor, Michigan, USA.; ^4^ Department of Psychiatry, Women's College Hospital, University of Toronto, Toronto, Canada.; ^5^ Department of Psychology and Gerontology Institute, Georgia State University, Atlanta, Georgia, USA.; ^6^ National Institutes of Health Library, Office of Research Services, Office of the Director, National Institutes of Health, Bethesda, Maryland, USA.; ^7^ Department of Obstetrics and Gynecology, Institute for Public Health, and Medicine (IPHAM)—Center for Health Equity Transformation, Northwestern University, Chicago, Illinois, USA.

**Keywords:** menopause transition, mental health, race, ethnicity, perimenopause, women

## Abstract

**Background:**

Racially and ethnically minoritized (REM) women experience social and structural factors that may affect their response to mental health treatment and menopausal symptoms during the menopause transition (MT). This scoping review on mental health during the MT for REM women in the United States was conducted to characterize factors associated with mental health challenges.

**Materials and Methods:**

Five databases were searched. Articles were included if focused on MT in REM women in the United States and its territories with specific mental illnesses and published in English from 2005 to 2021. Titles and abstracts and full text were screened. Screening and data collection were completed in duplicate by two reviewers in Covidence.

**Results:**

Sixty-five articles were included and indicate that REM women experience a disproportionate burden of depressive symptoms during the MT. Less evidence is reported about anxiety, Post-Traumatic Stress Disorder, psychosis, schizophrenia, and other mental illnesses. The risk factors associated with mental illness during MT are social, structural, and biological. Treatment response to therapeutic interventions is often underpowered to explain REM differences.

**Conclusion:**

Depression during the MT is associated with negative outcomes that may impact REM women differentially. Incorporating theoretical frameworks (*e.g.*, intersectionality, weathering) into mental health research will reduce the likelihood that scientists mislabel race as the cause of these inequities, when racism and intersecting systems of oppression are the root causes of differential expression of mental illness among REM women during the MT. There is a need for interdisciplinary research to advance the mental health of REM women.

## Introduction

Racial and ethnic inequities in health persist in the United States despite decades of research and programs. Structural racism is one upstream area that must be addressed to dismantle such inequities. Structural racism is defined as “the totality of ways in which societies foster racial discrimination through mutually reinforcing systems of housing, education, employment, earnings, benefits, credit, media, healthcare, and criminal justice.”^1^ There are other exposures such as socioeconomic status, experience of everyday discrimination, and life stress that Dr. David Williams depicts in his “house that racism built.”^2^ These structures create access barriers that then compound the disparities for racially and ethnically minoritized (REM) women who experience mental illness. REM women refers to those women historically marginalized by the majority (non-Hispanic White [NHW]) due to race, ethnicity, or both^3^ and includes Black/African American, Hispanic/Latina/ex, Native/Indigenous, and Asian and Pacific Islander.

The menopause transition (MT), “the period immediately before menopause (when the endocrinologic, biologic, and clinical features of approaching menopause commence) and the first year after menopause,”^4^ is an important journey in a woman's life. Over two million U.S. women enter menopause annually,^5^ including 460,000 REM women. Menopause symptoms impact at least 20% of all women, and a higher prevalence of psychiatric symptoms is reported among women going through a symptomatic MT.^6^ There is limited information on the cause of this higher prevalence of psychiatric symptoms in women in general and in REM women in particular. While studies show that mental health and menopause symptoms are inextricably linked and disproportionately impact REM women,^7^ the factors that contribute to this difference in prevalence of mental illness among REM women are understudied.

Therefore, this scoping review presents a synthesis of the findings from the literature on mental health research as it relates to REM women during the MT. The aim is to summarize the evidence from literature on mental health during the MT in REM women in the United States with a focus on vasomotor symptoms (VMS), quality of life, mental health outcomes, and clinical management and to identify research gaps for future research efforts.

## Materials and Methods

### Frameworks

Intersectionality and the weathering hypothesis were used to inform the research question, hone the eligibility criteria, and to identify research gaps for this scoping review.^8,9^

### Protocol and registration

We followed the scoping review methods from the Joanna Briggs Institute.^10^ A protocol was written *a priori* following the Preferred Reporting Items for Systematic Reviews and Meta-Analyses extension for Scoping Reviews (PRISMA-ScR) Checklist^11^ (Supplementary File S1), and the PRISMA-ScR was used for reporting of this review.

### Eligibility criteria

For inclusion, articles needed to include study findings on REM women during the MT where the age range fell within ages 48 and 58 years. Studies that included a mix of NHW and REM women were included. All studies must have been conducted in the United States, including its territories and published in English from 2005 to 2021. Studies were required to examine at least one of the following mental illnesses *or risk factors*: mood disorders, psychosis, generalized anxiety disorder, schizophrenia, substance use and co-occurring mental illnesses, obsessive compulsive disorder, bipolar disorder, disruptive mood dysregulation disorder, borderline personality disorder, family violence, intimate partner violence, and post-traumatic stress disorder (PTSD).

Articles were excluded if (1) REM women were not included in the study; (2) the article focused exclusively on women before or after the MT; (3) the research participants did not reside in the United States, including its territories; (4) the article was not in English; or (5) the article type was any type of review article, commentary, letter, conference abstract, errata, or corrigenda.

### Information sources and search strategy

The search strategy was developed by the biomedical librarian (A.A.L.) in consultation with two members of the review team (T.L.J. and L.M.R.). The search strategy was validated through the retrieval of a preidentified set of relevant studies and peer reviewed by another librarian. The biomedical librarian searched five databases: CINAHL Plus (Ebscohost, 1981–current), EMBASE (Elsevier, 1947–current), PsycNet (American Psychological Association, 1806–current), PubMed (U.S. National Library of Medicine, 1946–current), and Web of Science: Core Collection (Clarivate Analytics, 1900–current). The searches were limited to results published in English from 2005 to 2021. In addition, a search strategy was used to limit studies conducted in the United States or its territories as that was the study location of interest. Finally, we used search strategies (see Supplementary File S2) to remove animal studies and specific article types (*e.g.*, conference abstracts, letters, editorials, reviews) that were detailed in our exclusion criteria from search results.

A combination of keywords and controlled vocabulary search terms (*i.e.*, CINAHL Subject Headings, EMTREE, Medical Subject Headings [MeSH], Thesaurus of Psychological Index Terms) were used for each concept of interest: menopause, REM women menopause, and mental health conditions. The searches were conducted in February 2022. See Supplementary File S2 for comprehensive details on the search strategy.

For all included articles, the bibliographies were reviewed to identify other potentially relevant articles not identified in the original search. In addition, review team members who are experts in the field of health disparities research, women's health, psychiatry, and clinical psychology identified additional relevant published research articles for potential inclusion. All articles identified through these supplemental methods were further screened using the eligibility criteria.

All results from the database searches were exported into EndNote 20 (Clarivate Analytics) and duplicates identified. The unique records retrieved were then exported into Covidence (Veritas Health Innovations), which was used for study screening and data collection.

### Selection of sources of evidence

Before commencing screening, 6 reviewers conducted a pilot of the screening process at both levels using a random sample of 20 records selected by the biomedical librarian. After completing the screening pilot, the team discussed conflicts and clarified the eligibility criteria (see Supplementary File S1 for the protocol).

A two-step screening process was used. First, two reviewers independently screened the titles and abstracts using the eligibility criteria. Next, the full text of all articles included after title and abstract screening was independently screened using the same criteria. The six reviewers were divided into three pairs to screen the full text of articles to ensure that they met the eligibility criteria. A different third reviewer adjudicated discrepancies by reviewing the article and discussing the discrepancy with the two reviewers to arrive at a consensus for the article to be included or excluded.

### Data collection and data items

Covidence was used for data collection. A pilot of the data collection step was completed by L.M.R. and T.L.J. and revisions made to the data collection form in Covidence before commencing to further clarify the items to collect. Two reviewers (L.M.R., T.L.J.) collected data from each included article. One reviewer (T.L.J.) reconciled all discrepancies in Covidence from data collection.

The data collected included citation information, race and ethnicity, total sample by race and ethnic group and overall, study setting, state/city of study, study design, intervention tested, stage of MT, menopausal symptoms reported, mental health and other health conditions studied, study instruments used, if a secondary data set was used and from where, study outcomes, study outcomes by racial and ethnic group, limitations of study, funding source, and possible conflicts of interest. Excel (Microsoft) was used for data cleaning and analysis. Key characteristics were extracted from the data and are found in Table 1. Data are descriptively summarized in the Results and qualitatively synthesized in the Discussion section.

**Table 1.** Key Characteristics of Included Articles

Author (year)	Study setting	Stage of menopause reported	Sample size	Racial and ethnic groups included	Study design	Mental illness or symptoms reported	Menopause symptoms reported	Were race and ethnicity included in analysis?
Freeman et al. (2005)^39^	Pennsylvania	Stage of Reproductive Aging Workshop criteria (STRAW)	*N* = 436	African American, White	Cohort study—Penn Ovarian Aging Study (POAS)	Anxiety, Depressive symptoms	Hot flashes	Yes
Schnatz et al. (2005)^12^	Connecticut	Perimenopausal, Early postmenopausal	*N* = 80	Hispanic, Non-Hispanic (White, African American, Asian, Other)	Cross-sectional study	Depressive symptoms	Hot flashes, Night sweats, Sexual dysfunction, Vaginal dryness, Sleep quality, Insomnia	No
Suau et al. (2005)^27^	Puerto Rico	Menopausal, Premenopausal	*N* = 64	Hispanic (Puerto Rican, Dominican, Cuban, South American, Central American)	Cross-sectional study	Depressive symptoms	Not reported	Not applicable
Xu et al. (2005)^28^	Michigan	Perimenopausal, Early Postmenopausal, Premenopausal	*N* = 342	White, African American, Other, Not Reported	Cross-sectional study	Anxiety, Depressive symptoms, Irritability	Hot flashes, Night sweats, Sexual dysfunction, Vaginal dryness	Yes
Freeman et al. (2006)^13^	Pennsylvania	STRAW criteria	*N* = 231	African American, White	Cohort study—POAS	Depression, Depressive symptoms	Hot flashes, Sleep quality, Insomnia	No
Kravitz et al. (2006)^40^	California, Illinois, Massachusetts, Michigan, New Jersey, Pennsylvania	Perimenopausal, Premenopausal	*N* = 1,538	African American, Caucasian, Japanese, Chinese	Cohort study—Study of Women Across the Nation (SWAN)	Depressive symptoms	Hot flashes, Night sweats	Yes
Sajatovic et al. (2006)^29^	USA, Not Specified	Perimenopausal, Early Postmenopausal	*N* = 91	African American, White, Other	Cross-sectional study	Bipolar disorder, Depression, Schizophrenia	Vasomotor, Psychosocial, Physical, Sexual	Yes
Wang (2006)^30^	Florida	Perimenopausal, Early Postmenopausal, Premenopausal	*N* = 333	Hispanic, White non-Hispanic	Cross-sectional study	Anxiety, Depressive symptoms, Irritability	Hot flashes, Night sweats, Sexual dysfunction, Vaginal dryness, Sleep quality, Insomnia	Yes
Bromberger et al. (2007)^41^	California, Illinois, Massachusetts, Michigan, New Jersey, Pennsylvania	Perimenopausal, Early Postmenopausal, Premenopausal	*N* = 3,302	African American, Chinese, Hispanic, Japanese, White	Cohort study—SWAN	Depressive symptoms	Hot flashes, Night sweats	Yes
Freeman et al. (2007)^42^	Pennsylvania	STRAW criteria	*N* = 404	African American, White	Cohort study—POAS	Depressive symptoms	Hot flashes, Sexual dysfunction, Vaginal dryness, Sleep quality, Insomnia	Yes
Gallicchio et al. (2007)^31^	Maryland	Perimenopausal, Premenopausal	*N* = 634	Black, White, Other	Cross-sectional study	Depressive symptoms	Hot flashes, Sexual dysfunction, Vaginal dryness, Sleep quality, Insomnia	Yes
Haren et al. (2007)^24^	Missouri	Perimenopausal or Premenopausal, Postmenopausal	*N* = 244	Black or African American	Cross-sectional study	Depressive symptoms	Not reported	Not applicable
Matthews et al. (2007)^43^	California, Illinois, Massachusetts, Michigan, New Jersey, Pennsylvania	Perimenopausal, Early Postmenopausal, Premenopausal, surgical menopause, indeterminant	*N* = 3239	African American, Chinese, Hispanic, Japanese, White	Cohort study—SWAN	Depressive symptoms	Not reported	Yes
Torigoe and Brown (2007)^26^	Hawaii	Perimenopausal, Early Postmenopausal, Premenopausal	*N* = 74	Other: multiethnic	Cross-sectional—Hilo Women's Health Study	Other: mood; perceived stress	Hot flashes	Not applicable
Woods et al. (2007)^14^	Washington	Menopause Transition, Middle and Late perimenopausal, Postmenopausal	*N* = 41	African American Asian/Pacific Islander, White, Other: Hispanic, mixed	Cohort study—Seattle Midlife Women's Health Study (SMWHS)	Depressive symptoms	Hot flashes, Sexual dysfunction, Vaginal dryness, Sleep quality, Insomnia	No
Goldbacher (2007)^44^	Pennsylvania	Perimenopausal, Early Postmenopausal, Postmenopausal included hysterectomy, oophorectomy	*N* = 421	Black, White	Cohort study—SWAN	Anxiety, Depression	Not Reported	Yes
Nelson et al. (2008)^45^	Pennsylvania	Menopause transition, Early Postmenopausal, Premenopausal	*N* = 436	African American, Non-Hispanic White	Cohort study—Women living in Philadelphia	Anxiety, Depressive symptoms	Hot flashes, Night sweats, Sexual dysfunction, Vaginal dryness, Sleep quality, Insomnia	Yes
Pien et al. (2008)^46^	Pennsylvania	Menopause Transition, Early Postmenopausal, Premenopausal	*N* = 436	African American, White	Cohort study—POAS	Anxiety, Depressive symptoms	Hot flashes, Sleep quality, Insomnia	Yes
Thurston et al. (2008)^47^	Pennsylvania	Perimenopausal, Early Postmenopausal, Premenopausal, indeterminant	*N* = 332	African American, White	Cohort study—SWAN	Depressive symptoms, Other: Childhood trauma	Hot flashes, Night sweats	Yes
Thurston et al. (2008)^48^	California, Illinois, Massachusetts, Michigan, New Jersey, Pennsylvania	Perimenopausal, Early Postmenopausal, Premenopausal, indeterminant	*N* = 1042	African American, White, Chinese, Japanese	Cohort study—SWAN	Anxiety, Depressive symptoms, Irritability	Hot flashes, Night sweats, Sleep quality, Insomnia	Yes
Woods et al. (2008)^15^	Washington	Menopause Transition, Early Postmenopausal, Premenopausal	*N* = 302	African American Asian/Pacific Islander, White, Other: Hispanic, mixed	Cohort study—SMWHS	Depressive symptoms	Hot flashes	No
Avis et al. (2009)^49^	United States not specified	Perimenopausal, Early Postmenopausal, Surgical menopause at follow-up were not included in analysis	*N* = 2943	Caucasian, African American, Hispanic, Chinese, Japanese	Cohort study—SWAN	Depressive symptoms, Other: Stress	Hot flashes, Night sweats, Sexual dysfunction, Vaginal dryness, Sleep quality, Insomnia, Other: Leaking urine	Yes
Bromberger et al. (2009)^50^	Pennsylvania	Perimenopausal, Premenopausal	*N* = 266	African American, White	Cohort study—SWAN	Anxiety, Depression	Hot flashes, Night sweats	Yes
Woods et al. (2009)^16^	Washington	Menopause Transition, Early Postmenopausal, Late reproductive, menopausal transition stages—early and late	*N* = 418	African American, Asian/Pacific Islander, White, Other: Hispanic, mixed	Cohort study—SMWHS	Depressive symptoms	Hot flashes	No
Bromberger et al. (2010)^51^	California, Illinois, Massachusetts, Michigan, New Jersey, Pennsylvania	Perimenopausal, Early Postmenopausal, Premenopausal	*N* = 3296	African American, White, Chinese, Japanese, Hispanic	Cohort study—SWAN	Depressive symptoms	Hot flashes, Night sweats	Yes
Green et al. (2010)^52^	New Jersey	Premenopausal, Perimenopausal, and Postmenopausal	*N* = 419	Hispanic, White	Cohort study—SWAN	Anxiety, Depressive symptoms	Sleep quality, Insomnia	Yes
Greendale et al. (2010)^53^	California, Illinois, Massachusetts, Michigan, New Jersey, Pennsylvania	Perimenopausal, Early Postmenopausal, Premenopausal	*N* = 1903	African American, Caucasian, Chinese, Hispanic, Japanese	Cohort study—SWAN	Anxiety, Depressive symptoms, Irritability	Hot flashes, Night sweats, Sleep quality, Insomnia	Yes
Kornstein et al. (2010)^32^	41 primary or psychiatric care settings across the United States	Premenopausal, Perimenopausal Early Postmenopausal	*N* = 2652	Race: Black, White, Ethnicity: Hispanic, Not Hispanic	Cross-sectional study—secondary analysis of Sequenced Treatment Alternatives to Relieve Depression (STAR^*^D)	Depression, Depressive symptoms, Comorbidity: Anxiety, PTSD Obsessive Compulsive Disorder, social phobia, agoraphobia, somatoform, hypochondriasis, bulimia, and drug and alcohol abuse	Not reported	Yes
Matthews et al. (2010)^54^	California, Illinois, Massachusetts, Michigan, New Jersey, Pennsylvania	Perimenopausal, Early Postmenopausal, Hysterectomy, status unknown	*N* = 1781	African American, White, Chinese, Hispanic, Japanese	Cohort study—SWAN	Depressive symptoms	Sleep problems	Yes
Rebbeck et al. (2010)^55^	Pennsylvania	Menopause Transition, Early Postmenopausal, Premenopausal	*N* = 436	African American, White	Cohort study—POAS	Depressive symptoms	Hot flashes	Yes
Schnatz et al. (2010)^56^	Connecticut	Perimenopausal, Postmenopausal	*N* = 102	Hispanic, Non-Hispanic, Ethnicity not reported	Cohort study—Women's Life Center at Hartford Hospital	Anxiety, Depression	Sexual dysfunction, Vaginal dryness, Sleep quality, Insomnia	Yes
Seritan et al. (2010)^33^	California	Perimenopausal, Early Postmenopausal, Premenopausal	*N* = 487	African American, White, Asian, Hispanic	Cross-sectional study	Anxiety, Depressive symptoms	Hot flashes, Night sweats	Yes
Wheatley (2009)^17^	Florida	Perimenopausal, Postmenopausal	*N* = 206	African American	Cross-sectional study	Depressive symptoms	Hot flashes, Sexual dysfunction, Vaginal dryness, Sleep quality, Insomnia	No
Bromberger et al. (2011)^57^	Pennsylvania	Perimenopausal, Menopause Transition, Early Postmenopausal Premenopausal	*N* = 221	African American, Caucasian	Cohort study—SWAN	Depression, Depressive symptoms	Hot flashes, Night sweats	Yes
Kravitz et al. (2011)^58^	California, Illinois, Michigan, Pennsylvania	Perimenopausal, Early Postmenopausal	*N* = 343	African American, Caucasian, or White, Chinese	Cohort study—SWAN	Anxiety, Depressive symptoms	Hot flashes, Night sweats	Yes
Morrison et al. (2011)^59^	Pennsylvania	STRAW criteria	*N* = 436	African American, White	Cohort study—POAS	Depression, Depressive symptoms	Not reported	Yes
Woods et al. (2011)^18^	Washington	Menopause Transition, Early Postmenopausal	*N* = 184	African American, Asian/Pacific Islander, White, Other: Hispanic, mixed	Cohort study—SMWHS	Anxiety, Depressive symptoms	Hot flashes, sleep quality, Insomnia	No
Bromberger et al. (2012)^60^	Illinois, New Jersey, Pennsylvania	Perimenopausal Premenopausal	*N* = 934	African American, Hispanic, White,	Cohort study—SWAN	Depression, Other: Premenstrual symptoms (mood)	Not reported	Yes
Gibson et al. (2012)^61^	California, Illinois, Massachusetts, Michigan, New Jersey, Pennsylvania	Perimenopausal Premenopausal, hysterectomy with ovarian conservation, reported hysterectomy with bilateral oophorectomy	*N* = 1970	African American, Hispanic, Asian, White	Cohort study—SWAN	Anxiety, Depressive symptoms	Not reported	Yes
Brandon et al. (2013)^19^	Texas, Pennsylvania	Perimenopausal, Early Postmenopausal Premenopausal, surgical menopause	*N* = 355	White, Black, Hispanic, Other	Nonrandomized Clinical Trial	Depression, Depressive symptoms	Not reported	No
Bromberger et al. (2013)^62^	California, Illinois, Massachusetts, Michigan, New Jersey, Pennsylvania	Perimenopausal, Early Postmenopausal, Premenopausal, postmenopausal, and hormone therapy	*N* = 2956	African American, White, Chinese, Hispanic, Japanese	Cohort study—SWAN	Anxiety	Hot flashes, Night sweats	Yes
Greenblum et al. (2013)^20^	Florida	Menopause Transition, Early Postmenopausal	*N* = 112	White, Hispanic, African American, Asian	Cross-sectional study	Anxiety, Irritability	Hot flashes, night sweats, sexual dysfunction, Vaginal dryness, sleep quality, Insomnia	No
Kornstein et al. (2013)^21^	41 primary or psychiatric care settings across the United States	Premenopausal, Perimenopausal, Postmenopausal	*N* = 1883	Race: Black, White, Ethnicity: Hispanic, Not Hispanic	Clinical trial—secondary analysis of STAR^*^D	Depression, Depressive symptoms	Not reported	No
Appelhans et al. (2014)^63^	California, Illinois, Massachusetts, Michigan, New Jersey, Pennsylvania	Perimenopausal, Early Postmenopausal, Premenopausal, undetermined, and surgical menopause	*N* = 3220	African American, White, Chinese, Hispanic, Japanese	Cohort study—SWAN	Depressive symptoms	Not reported	Yes
Colvin et al. (2014)^64^	Pennsylvania	Perimenopausal, Premenopausal	*N* = 303	African American, White	Cohort study—SWAN	Anxiety, Depression	Hot flashes, Night sweats	Yes
Kravitz et al. (2014)^65^	Pennsylvania	Perimenopausal, Premenopausal	*N* = 425	Black, White	Cohort study—SWAN	Anxiety, Depression, Depressive symptoms	Hot flashes, Night sweats	Yes
Richard et al. (2014)^34^	USA Not Specified	Perimenopausal	*N* = 193	White, Black, Mexican American, Other	Cross-sectional study—National Health and Nutrition Survey	Depressive symptoms	Not reported	Yes
Avis et al. (2015)^66^	California, Illinois, Massachusetts, Michigan, New Jersey, Pennsylvania	Perimenopausal, Early Postmenopausal, Premenopausal	*N* = 1,449	African American, White, Chinese, Hispanic, Japanese	Cohort study—SWAN	Anxiety, Depressive symptoms, Other: Perceived stress	Hot flashes, Night sweats	Yes
Dugan et al. (2015)^67^	California, Illinois, Massachusetts, Michigan, New Jersey, Pennsylvania	Perimenopausal, Early Postmenopausal	*N* = 2,891	African American, White, Chinese, Hispanic, Japanese	Cohort study—SWAN	Depressive symptoms	Hot flashes, Night sweats	Yes
Im et al. (2015)^35^	United States Not specified	Perimenopausal, Early Postmenopausal, Premenopausal	*N* = 542	African American, White, Hispanic, Asian	Cross-sectional study—secondary analysis of a large internet survey study	Depressive symptoms	Not reported	Yes
Prairie et al. (2015)^68^	California, Illinois, Massachusetts, Michigan, New Jersey, Pennsylvania	Perimenopausal, Early Postmenopausal, Premenopausal, surgical postmenopausal, undeterminable	*N* = 1716	African American, White, Chinese, Japanese, Hispanic	Cohort study—SWAN	Depressive symptoms	Sexual dysfunction, Vaginal dryness, Sleep quality, Insomnia	Yes
Freeman et al. (2016)^69^	Pennsylvania	Straw criteria	*N* = 436	African American, Black, Caribbean, Continental African, White, Non-Hispanic	Cohort study—POAS	Anxiety, Depression, Irritability, Mood deterioration	Hot flashes, Night sweat, Sexual dysfunction, Vaginal dryness Sleep quality, Insomnia	Yes
Keshishian et al. (2016)^22^	United States Not specified	Menopause symptoms diagnosis and/or hormone therapy claim, No menopause symptoms and/or a hormone therapy claim	*N* = 142,152	Black, White, Hispanic, Asian, American Indian or Alaskan Native, Native Hawaiian or Other Pacific Islander, Other	Cross-sectional study—U.S. national database	Anxiety, Depression	Sleep quality, Insomnia	No
Colvin et al. (2017)^70^	Pennsylvania	Perimenopausal, Early Postmenopausal, Premenopausal	*N* = 303	African American, Black, Caribbean, Continental African, White, Non-Hispanic	Cohort study—SWAN	Depression	Hot flashes, Night sweats	Yes
Marsh et al. (2017)^71^	California, Illinois, Massachusetts, Michigan, New Jersey, Pennsylvania	Perimenopausal, Menopause Transition, Early Postmenopausal	*N* = 1,306	African American, White, Chinese, Hispanic, Japanese	Cohort study—SWAN	Depressive symptoms	Hot flashes, Night sweats	Yes
Gordon et al. (2018)^76^	North Carolina	STRAW criteria	*N* = 172	African American, White	Randomized Clinical Trial	Depressive symptoms	Hot flashes, Night sweats	Yes
Bromberger et al. (2019)^72^	California, Illinois, Massachusetts, Michigan, New Jersey, Pennsylvania	Perimenopausal, Premenopausal	*N* = 3,300	White, Black, Japanese, Chinese Hispanic	Cohort study—SWAN	Depressive symptoms	Hot flashes, Night sweats, Sleep quality, Insomnia	Yes
Gibson et al. (2019)^73^	California	Perimenopausal, Menopause Transition, Postmenopausal, hormone therapy	*N* = 2,016	White, Black or African American, Latina or Hispanic	Cohort study—Reproductive Risks of Incontinence Study at Kaiser	Anxiety, Depressive symptoms, PTSD	Hot flashes, Night sweats, Sexual dysfunction, Vaginal dryness, Sleep quality, Insomnia	Yes
Dickins et al. (2020)^23^	Massachusetts	Perimenopausal	*N* = 33	Black or African American, White, American Indian, More than one race, Hispanic	Cross-sectional study—Secondary data analysis of a study of menopause and health symptoms in women with and without HIV	Anxiety, Depression	Sleep quality, Insomnia	No
Im et al. (2020)^36^	USA Not Specified	Perimenopausal, Early Postmenopausal, Premenopausal	*N* = 1,054	African American, White, Hispanic, Asian	Cross-sectional study—Secondary analysis of two larger internet survey studies	Depressive symptoms	Sleep quality, Insomnia	Yes
Jones et al. (2020)^74^	California	Perimenopausal, Early Postmenopausal, Premenopausal	*N* = 264	African American, Latina, White	Cohort study—Midlife Women's Health Study	Depressive symptoms	Hours of sleep (self-reported)	Yes
Still et al. (2020)^25^	Midwest, USA	Perimenopausal, Menopausal	*N* = 184	African American, Black, Caribbean, Continental African	Cross-sectional study—Secondary analysis of a large multisite study	Depression	Hot flashes, Sexual dysfunction, Vaginal dryness, Sleep quality, Insomnia	Not applicable
De Mello et al. (2021)^37^	Arizona	Perimenopausal, Early Postmenopausal	*N* = 255	African American, Asian, Hispanic, White, Other	Cross-sectional study	Anxiety, Depressive symptoms	Hot flashes, Sexual dysfunction, Vaginal dryness	Yes
de Wit et al. (2021)^75^	Massachusetts	STRAW criteria	*N* = 50	Black, White, Native American, Asian	8-week Observational study	Depressive symptoms, Irritability	Vasomotor symptom frequency	Yes
Ji et al. (2021)^38^	Texas	Menopausal	*N* = 384	Black, White, Hispanic	Cross-sectional study—Secondary analysis of the Dallas Heart Study	Depressive symptoms	Hot flashes, Sexual dysfunction, Vaginal dryness	Yes

### Patient and public involvement

While neither patients nor the public were involved in this scoping review, the results may be of interest and most generalizable to persons who identify as REM women, their clinicians, researchers, and funders in that knowledge gaps are identified, and future areas of research highlighted in the Discussion section.

## Results

**FIG. 1.**
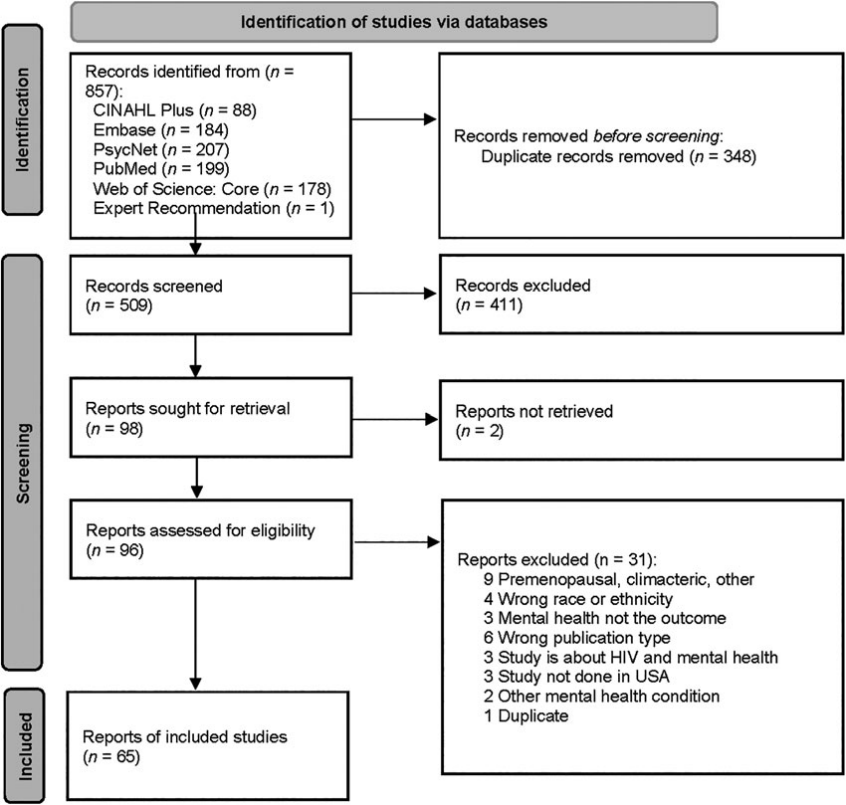
PRISMA Flow Diagram.

A total of 857 articles were retrieved from the 5 databases searched, including 1 article recommended by team members, of which 348 were duplicates and 509 were unique. Of the 509 screened at title and abstract level, 411 were excluded and 96 proceeded to full text review. After full text screening, 65 articles were included in the review and 31 were excluded (Fig. 1).

### Study characteristics

Sixty-five studies were reported on mental illnesses during the MT among REM women. The key characteristics of race, ethnicity, sample size, mental health symptoms or illnesses, stage of MT, menopause symptoms, study design, and study settings are presented in Table 1.

#### Race and ethnicity

Twelve studies^12–23^ did not include race or ethnicity in the statistical analysis, and three studies included only Black, African American, Caribbean, or Continental African women,^17,24,25^ one included only “multiethnic” women from Hilo, Hawaii,^26^ and one included only Hispanic women.^27^

#### Study design

There were 20 cross-sectional,^12,17,20,22–38^ 41 cohort,^13–16,18,39–74^ 1 observational,^75^ and 3 clinical trial studies.^19,21,76^ Of the cohort studies, 26 were from the Study of Women Across the Nation (SWAN),^40,41,43,44,47–54,57,58,60–68,70–72^ 7 from the Penn Ovarian Aging Study (POAS),^13,39,46,55,59,69^ 4 from the Seattle Midlife Women's Health Study (SMWHS),^14–16,18,74^ 1 from the Midlife Women's Health Study,^74^ 1 from the Reproductive Risks of Incontinence Study at Kaiser,^73^ 1 from data collected at Women's Life Center at Hartford Hospital,^56^ and 1 from data collected from women in Philadelphia.^45^

#### Study setting

Thirty-nine studies^12–18,20,23,24,26–28,30,31,33,37–39,42–47,50,52,55–57,59,64,65,69,70,73–76^ used a state or U.S. territory as the study setting. Eighteen studies^19,25,40,41,47,51,53,54,58,60–63,66–68,71,72^ used multiple states as the study setting, and eight studies^21,22,29,32,34–36,49^ used a nationwide study setting. The SWAN and POAS cohort study data recruited women from urban and suburban areas. Sample Size:The sample sizes ranged from 33 to 142,152, with the largest using data from a U.S. national database.

#### Stage of MT

Seven studies used the Stages of Reproductive Aging Workshop to determine the stage of MT,^13,39,42,59,69,75,76^ seven studies included surgical menopause, hysterectomy, or oophorectomy in the definition of the stage of MT,^19,43,44,49,54,61,63^ and the rest defined the menopausal stage by menstrual bleeding patterns, timing of last menstrual period, or midlife age range.

#### Mental illness or symptoms

The majority (63 of 65) of studies reported findings on depressive symptoms or major depression. This was followed by 26 studies on anxiety,^18,20,22,23,28,30,32,33,37,39,44–46,48,50,52,53,56,58,60,61,64–66,69,73^ and 7 assessing irritability.^20,28,30,48,53,69,75^ Three studies reported findings on serious mental illnesses such as PTSD, bipolar disorder, or schizophrenia.^29,32,73^

#### Menopausal symptoms

The majority of studies reported on menopause symptoms such as vasomotor or sleep problems (52 of 65) and how these related to mental health and quality of life. Of the three clinical trials, one was a double-blinded, placebo-controlled trial of estradiol and progesterone,^76^ one was a nonrandomized trial of cognitive therapy (CT),^19^ and one was a secondary analysis of Sequenced Treatment Alternatives to Relieve Depression (STAR*D)^21^ with only one^76^ of these studies, including race/ethnicity in the analysis.^76^

## Discussion

### Mental health outcomes during MT

The MT is a period of increased vulnerability to negative mental health outcomes, particularly depression. Studies that include REM women report prevalence rates of depressive symptoms ranging from 16.5% to 28%^31,41,68,74^ or major depression ranging from 11% to 15.8%.^50,60^ A 10-year study found a two- to four-fold increase in risk for a major depressive episode in women who were in the MT or postmenopausal compared to women who were premenopausal.^57^ Similar findings have been reported by other studies.^13,25,41,42,51^ In general, studies show that the risk is higher in the MT than in postmenopause,^33^ with some suggestion that late MT is the most vulnerable period.^15,70^ However, despite this consensus, some studies have found either no relationship between menopausal status and depressive symptoms or lower depressive symptoms in the MT compared to premenopause.^31,59,61^ These discrepancies were attributed to differences in subject samples (*e.g.*, inclusion of only women without a history of mental health problems, and not taking psychotropic medication).

There are exceptions,^12,38,74^ but prevalence rates tend to be higher in Black and Hispanic women during MT.^30,41,51^ For example, in the SWAN study the prevalence rate for an episode of major depression over 10 years of follow-up was 33.8% for Black women compared to 29.9% for NHW women,^57^ and another SWAN report showed that Black women are 71% more likely to have an onset of depression over 7 years of follow-up.^50^ Higher rates of depression in Black compared to NHW women have been reported in other cohorts as well.^34^ Despite these relevantly consistent findings, there is evidence that Black women are less likely than NHW women to have a trajectory of increasing depressive symptoms over time, although the effect did not remain significant after controlling for education, financial strain, smoking, and bodily pain.^72^ Studies generally show higher rates of depression in Hispanic compared to NHW women.^52,71,77^ A longitudinal study reported that Hispanic women were more likely than NHW women to have a trajectory of increasing depressive symptoms over time.^72^ There is evidence of variability within Hispanic women. One study reported higher depressive symptoms in Puerto Rican women than in Dominican, Cuban, and South American women.^52^

Depression is associated with several negative outcomes. In addition to more severe menopausal symptoms such as hot flash bother^48^ and decreased sexual desire,^14^ negative physical outcomes include metabolic syndrome^44^ and greater waist circumference,^44^ particularly when depression is experienced in combination with nonemployment.^63^ Depression is associated with sleep disruption,^14^ higher levels of stress,^16^ and risk for other mental health symptoms, such as drug and alcohol misuse and anxiety.^44^ There is also evidence of depression-related cognitive dysfunction such as difficulty concentrating^14^ and slower cognitive processing speed.^53^ Some of the negative correlates appear to disproportionately impact Black and Hispanic women. For example, one study reported that Black and Hispanic women were more likely than NHW women to report functional disability,^49^ and another study found that sleep disruption was more strongly related to depression in Hispanic women.^72^ However, another study that examined depressive symptoms and sleep related symptoms among REM women in the MT found that total number of depressive and sleep related symptoms were highest among Asian women followed by Hispanic and Black women. Asian women were more likely to experience depression than other REM women but were frequently underreported, underdiagnosed, and undertreated for depressive and sleep related symptoms during midlife.^36^

Symptoms of anxiety may also increase during the MT,^62^ but the evidence for REM differences is mixed. Higher anxiety has been reported in Hispanic and Black women compared to NHW women.^62^ However, one study showed increasing anxiety in NHW but not Black women,^28^ while another found the opposite.^61^ Within Hispanic women, Dominican women report more trait anxiety than South American and Cuban women. Risk factors for anxiety include more severe menopausal symptoms,^33,39,62^ sexual dysfunction,^56^ financial strain and other stressors,^23,62^ previous anxiety,^62^ lower education,^62^ and poor health.^62^ Anxiety symptoms are associated with reduced quality of life,^20^ increases in blood pressure,^26^ reduced memory and slower processing speed,^53^ and a higher risk for depression.^65^

There is less research on other mental illnesses during the MT. Potential reasons for the gap in research include but are not limited to the possibility that women with other mental illnesses may be lost to follow-up, the lack of targeted sampling of women with other mental illnesses, or exclusion criteria that prohibited women with serious mental illness from participating in clinical trials. A few studies suggest that lifetime history of interpersonal violence and sexual assault, childhood abuse or neglect, and current PTSD are associated with menopause symptoms.^47,73^ Women with serious mental illness (schizophrenia or schizoaffective disorder, bipolar disorder, and major depression) report feeling depressed, anxious, tired or worn out, and lacking in energy, and report experiencing poor memory.^29^

### VMS impact on mental health and quality of life

Although most women experience some degree of VMS during the MT, Black women report the longest duration (median 10.1 years) of VMS compared to NHW, Chinese, Japanese, and Hispanic women.^66^ Risk factors for longer duration of VMS include younger age, lower educational level, greater perceived stress, and symptom sensitivity.^66^ Self-reported depressive and anxiety symptoms were associated with bothersomeness of VMS.^33^ Findings from the SWAN Mental Health study found a history of childhood abuse, and neglect was associated with increased VMS. When comparisons of NHW and Black women with a past history of child abuse and neglect and VMS were conducted, the findings were not statistically significant but did suggest a stronger association in Black women.^47^ When researchers controlled for frequency of VMS, Black women were more bothered by VMS than NHW and Japanese women but the reason for REM differences is understudied.^48^

Quality of life during the MT includes a complex array of factors. A study of midlife women found that sleep disturbances, fatigue, and anxiety most significantly affected quality of life.^20^ Lower health related quality of life, reduced role functioning, more negative appraisal of aging, and perceived poorer health were higher in women with depression.^16,49,66^ African American and Hispanic women reported more bodily pain and reduced social functioning that NHW.^49^

### Clinical management of mental health during MT

Treatments that specifically target mood disorders, anxiety, and psychosis associated with the MT have not been developed. However, several investigators have found that standard treatments for depression and anxiety are effective for some women in the MT. Expert consensus guidelines recommend selective serotonin reuptake inhibitors (SSRIs), serotonin and norepinephrine reuptake inhibitors (SNRIs), and psychotherapy as first-line treatments.^78^ While estrogen-based therapies have not been FDA approved for depression, there is evidence to suggest that estrogen has similar antidepressant effects to SSRIs and SNRIs for women during the MT but not postmenopause.^79,80^ Estrogen therapies^78^ have been shown to be most effective for treatment of depression as a monotherapy or augmentation to antidepressant treatment for women during the MT.

Despite the reported higher prevalence of depression in Black and Hispanic women, few studies specifically address feasibility, accessibility, and efficacy of frontline treatments for MT associated mood disorders among REM women. No studies addressed treatment of symptoms specifically due to psychosis, anxiety, or PTSD, known risk factors that are highly prevalent for REM women. Among the two studies^19,21^ of frontline treatments for depression during MT, treatment rates were not detailed by race or ethnicity. The impact of secondary or other specific interventions (*i.e.*, taking hormonal therapy, exercising more than the recommended physical activity guidelines) on the outcome of depressive symptoms was explored in two studies^32,67^ but only included small samples of REM groups with the intervention (31% and 16.8%, respectively). One study, a randomized double-blinded, placebo trial of estradiol and progesterone, included similar sample sizes of REM (85%) to White (81%) women in the treatment intervention group.^76^ All, but one study,^35^ which assessed views on exercise and associated depressive symptoms, fell short of comparing REM and NHW women. In addition, receptiveness and accessibility to treatment were understudied.

Data from the STAR*D study were analyzed to understand if depression in women differs by stage of menopause or hormone therapy. The results from this analysis showed that women taking hormone therapy were significantly less likely to be Hispanic, Black, or of another race and were more likely to be NHW, more educated, married, and privately insured. Women taking hormone therapy were more likely to have a recurrent course of depression along with increased medical comorbidities. This analysis showed that women taking hormone therapy reported better physical functioning, fewer melancholic features, and less sympathetic arousal compared with women not taking hormone therapy.^32^

Exercise has been shown to be part of a combination of effective treatments for mood disorders during the MT. A study of REM women (Hispanic [23.4%], Asian [22.7%], Black [24.9%], and NHW [29.0%]) found that depressive symptoms negatively correlated with active living and exercise physical activities and positively correlated with occupational physical activities. Race was not associated with the magnitude of exercise related improvement in mood.^35^ Analysis of SWAN data found that physical activity was associated with lower risk of high depressive symptoms (*i.e.*, CES-D score of 16 or higher) across 10 years. Japanese and Hispanic women reported higher depressive symptoms compared to NHW women. Whether exercise effectively reduces high depressive symptoms for Japanese or Hispanic women was not discussed.^67^

Among psychotherapies, CT has the most evidence for effective treatment of mood during the MT. A study of the effectiveness of CT in women who were not taking or had discontinued psychotropic medication indicated that greater than one-half (55%) of the women, despite stage of menopause, had a reduction in symptoms. In addition, the rate of early and late response, as well as noncompletion and completion of CT, was assessed but didn't compare differences in these categories for women of varying racial backgrounds.^19^

#### Research gaps (translational, intervention, and services research)

This scoping review identified several research gaps on mental illness during the MT in REM women. Approximately 25% of the studies did not consider REM group in the statistical analysis. Another gap was the lack of large enough sample sizes to explain REM differences in studies that investigated treatment responses to therapeutic interventions for mental health symptoms. This highlights the necessity of sample sizes sufficiently powered to analyze data by REM group for future studies.

Health care services delivery, prevention intervention, intersectional research, mixed methods, and implementation science approaches are also absent from this body of research. Gender identity and sexual orientation were also not considered in this research. Incorporating research that includes gender identity and sexual orientation data will strengthen mental health research during the MT. Most studies did not consistently report the use of hormone therapy by study participants and its effect on mental health outcomes. Improved reporting on use of hormone therapy among REM women will advance this area of research. Most studies did not include American Indian, Alaskan Native, Native Hawaiian, and Pacific Islander women. Finally, the current literature does not address structural racism and as such scientists often mislabeled race as the cause of inequities when racism and intersecting systems of oppression are the root causes. Each of these topics deserves prospective research.

## Limitations

Although the highest rates of suicide for women occur during ages 45–64 years,^81^ coinciding with the MT, no articles on suicidal ideation were included in this scoping review. Although mental illness can contribute to a heightened risk for suicidal thoughts and behavior, suicide is often a response to a stressful life event that can include social, cultural, and economic factors as well. Future research is needed to examine suicidal behavior during MT with a focus on REM women.

## Conclusions

To our knowledge, this is the first scoping review on mental health during the MT among REM women living in the United States. These findings reflect that mental illness among REM women is understudied. Nonetheless, there is an opportunity to improve mental health research by putting REM women at the center of research through an integration of societal, economic, cultural, and biological factors and the use of research methods that advance a better understanding of mental illness among REM women. This transformative research requires multidisciplinary science that advances multilevel interventions grounded in local, state, and national policy efforts to improve mental health outcomes for REM women.

## Supplementary Material

Supplemental data

Supplemental data
