# Supplementary material for: Key Findings from Mental Health Research During the Menopause Transition for Racially and Ethnically Minoritized Women Living in the United States: A Scoping Review
Source: J Womens Health (Larchmt). 2024 Feb 13;33(2):113–31. doi: 10.1089/jwh.2023.0276 (PMC10880275; doi:10.1089/jwh.2023.0276)
Supplement: Supplemental data [file Supp_FileS1.docx]

**Key Findings from Mental Health Research during the Menopause Transition for Racial and Ethnically Marginalized Women: A Scoping Review Protocol**

**Authors:**

Tamara Lewis Johnson

Women’s Mental Health Research Program, Office of Disparities Research and Workforce Diversity, National Institute of Mental Health, NIH, Rockville, MD USA

Laura Rowland, PhD

Neuroscience of Mental Disorders and Aging Program, National Institute of Mental Health, NIH, Rockville, MD USA

Alicia A. Livinski, MPH, MA

National Institutes of Health Library, Office of Research Services, Office of the Director, National Institutes of Health, Bethesda, MD, USA [alicia.livinski@nih.gov](mailto:alicia.livinski@nih.gov)

**Rationale**

The rationale for the review is to: map key concepts that underlie mental health research during the menopause transition for racially and ethnically marginalized women (REMW); to clarify working definitions in the field of mental health research and to clarify the conceptual boundaries of the mental health research during the menopause transition for REMW in the USA.

**Objectives**

• What is a useful way to map and summarize the evidence from the research literature on mental illness during the menopause transition for racially and ethnically marginalized women in the USA?

• What are the research gaps in the mental health research literature during menopause from racially and ethnically marginalized women in the USA?

• How best to inform future mental health research for racially and ethnically marginalized women in the USA?

The objective is to provide a scoping review of the literature of the field of mental health research during the menopause transition for racially and ethnically marginalized women in the USA and to highlight the need for further research in this area.

**Protocol and registration**

The protocol will be posted to Open Science Framework.

**Eligibility criteria**

Inclusion criteria:

• Language: English only

• Publication year: 2005–2022

• Population: Black, Latina/ex, Biracial, Native/Indigenous, Asian women [if mixed population, including white, include at title/abstract screening step]

• Menopause (midlife, perimenopause, premature) transition

• Geography: US only

• Mental health condition including: mood disorders, psychosis, generalized anxiety disorder, schizophrenia, substance use and co-occurring mental disorders, obsessive compulsive disorder, bipolar disorder, disruptive mood dysregulation disorder, borderline personality disorder, family violence, intimate partner violence, and PTSD

• Publication/article type: research article, review articles, published dissertations/theses

Exclusion criteria:

• Language: not in English

• Population & Exposure: Not women in the menopause transition (midlife, perimenopause), or only women not considered Black, Latina/x, Biracial, Native/Indigenous, Asian

• Geography: studies where the research participants are not in the USA

• Outcome: not the specified mental health condition listed in the inclusion criteria

• Publication/article type: commentaries, reports, unpublished dissertations, and unpublished theses; conference abstracts/proceedings; protocols; letters, protocols, erratum, corrigendum

• Study design: studies about autism or attention deficit disorder or HIV and mental health

**Information Sources & Search**

A biomedical librarian will conduct the literature searches.

The citation and abstract databases: PubMed (US National Library of Medicine), Embase (Elsevier), PsycNet (American Psychological Association), Web of Science: Core Collection (Clarivate Analytics) and CINAHL Plus (Ebscohost) will be searched.

The citation and abstract database searches will be limited to articles published in English and within the last 15 years (2005–2022).

The reference lists of all included articles and relevant reviews will be screened by the authors to identify other potentially relevant articles. These articles will then be added to the total pool of articles and passed through the screening process.

EndNote 20 will be used to collect and manage the database search results.

A combination of keywords and controlled vocabulary terms (i.e., CINAHL Subject Headings, MeSH, EMTREE, Thesaurus of Psychological Index Terms) will be used for each concept of interest: menopause; racial and ethnic marginalized women; and mental health conditions. The search terms were reviewed by the review team for feedback prior to being finalized by the biomedical librarian.

**Selection of Sources of Evidence**

The online tool, Covidence (Veritas Health Innovations) will be used for the screening (i.e., selection of sources) step.

A two-step screening process will be used. First, two reviewers will independently screen each article by reading the title and abstract and using the eligibility criteria to include or exclude the article. Disagreements between the two reviewers will be resolved by consensus or by a third reviewer.

Second, all articles included during the title and abstract screening step will proceed to the full text review step. Two reviewers will independently screen each article using the eligibility criteria and full text of the article. Disagreements between the two reviewers will be resolved by consensus or by a third reviewer.

A pilot will be conducted with all reviewers prior to commencing the formal review in order to test out and adapt the eligibility criteria and screening process.

**Data Charting Process**

The data collection will be done using Covidence by two reviewers independently collecting the data from each included article. A third reviewer will compare the collected data to identify and resolve discrepancies between the reviewers.

Missing or unclear data will be marked as missing or not reported.

Multiple articles reporting the same study will be grouped together and the data collected as a block by the two reviewers.

**Data items**

The data items collected included: citation information, race/ethnicity, total sample by race/ethnic group/overall, study setting, state/city of study, study design, intervention tested, stage of menopause transition, menopausal symptoms reported, mental health and other health conditions studied, study instruments used, if a secondary data set used and from where, study outcomes, study outcomes by racial/ethnic group, limitations of study, funding source, and possible conflicts of interest.

**Synthesis of results**

Results will be summarized and charted by different REMW (race, ethnicity), mental disorder (SMI, psychosis, mood disorders, STB) and structural factors (housing, educational attainment, wage inequities, language, culture), policy and clinical practice.

**Funding**

This work was completed as part of the NIH Intramural Research Program and official NIH duties (LR, TLJ, AAL).
