# Supplementary material for: Key Findings from Mental Health Research During the Menopause Transition for Racially and Ethnically Minoritized Women Living in the United States: A Scoping Review
Source: J Womens Health (Larchmt). 2024 Feb 13;33(2):113–31. doi: 10.1089/jwh.2023.0276 (PMC10880275; doi:10.1089/jwh.2023.0276)
Supplement: Supplemental data [file Supp_FileS2.docx]

**Supplemental File 2: Final search strategies used for literature searches**

**Database:** PubMed

**Vendor:** US National Library of Medicine

**Date of search:** February 8, 2022

**Limits:** Language: English; Publication date: 1/1/2005–12/31/2021; Publication/Article type: exclude letter, comment, editorial, retracted publication, meeting abstract, conference abstracts. Species: exclude animal studies.

| **Concept** | **Search Terms Used** |
| --- | --- |
| **Menopause** | (menopause[tiab] OR menopausal[tiab] OR “peri-menopaus*”[tiab] OR perimenopaus*[tiab] OR "Menopause"[mesh:noexp] OR "Menopause, Premature"[Mesh] OR "Perimenopause"[Mesh]) |
| **Mental Health Conditions** | AND (“mental illness*”[tiab] OR “mental disorder*”[tiab] OR “mental health”[tiab] OR “mentally ill”[tiab] OR “mood disorder*”[tiab] OR “affective disorder*”[tiab] OR depression[tiab] OR depressed[tiab] OR “depressive disorder*”[tiab] OR psychosis[tiab] OR psychoses[tiab] OR “psychotic disorder*”[tiab] OR “schizoaffective disorder*”[tiab] OR anxiety[tiab] OR “panic disorder*”[tiab] OR phobia*[tiab] OR “phobic disorder*”[tiab] OR schizophren*[tiab] OR bipolar[tiab] OR “manic disorder*”[tiab] OR “manic depressive”[tiab] OR “obsessive compulsive disorder*”[tiab] OR “disruptive mood dysregulation disorder*”[tiab] OR “borderline personality disorder*”[tiab] OR “Dysthymic Disorder*”[tiab] OR “Cyclothymic Disorder*”[tiab] OR “family violence”[tiab] OR “domestic violence”[tiab] OR “spousal abuse”[tiab] OR “spouse abuse”[tiab] OR “domestic abuse”[tiab] OR “elder abuse”[tiab] OR “child abuse”[tiab] OR “partner violence”[tiab] OR “battered woman”[tiab] OR “battered women”[tiab] OR PTSD[tiab] OR “post traumatic stress disorder*”[tiab] OR “posttraumatic stress disorder*”[tiab] OR “premenstrual dysphoric disorder*”[tiab] OR “premenstrual dysphoric syndrome*”[tiab] OR “pre-menstrual dysphoric disorder*”[tiab] OR “co occurring substance use”[tiab] OR “cooccurring substance use”[tiab] OR “cognitive decline*”[tiab] OR “cognitive dysfunction*”[tiab] OR “cognitive impairment*”[tiab] OR “Mild Neurocognitive Disorder*”[tiab] OR “suicidal ideation*”[tiab] OR “suicidal thought*”[tiab] OR “suicidal behavior*”[tiab] OR “suicide ideation*”[tiab] OR “sexual abuse”[tiab] OR “physical violence”[tiab] OR “physical abuse”[tiab] OR "Mental Health"[Mesh] OR "Mental Disorders"[Majr:NoExp] OR "Stress Disorders, Post-Traumatic"[Mesh] OR "Mood Disorders"[Mesh:NoExp] OR "Bipolar Disorder"[Mesh] OR "Anxiety Disorders"[Mesh] OR "Schizophrenia"[Mesh] OR "Psychotic Disorders"[Mesh] OR "Borderline Personality Disorder"[Mesh] OR "Domestic Violence"[Mesh] OR "Intimate Partner Violence"[Mesh] OR "Premenstrual Dysphoric Disorder"[Mesh] OR "Battered Women"[Mesh] OR "Depression"[Mesh] OR "Depressive Disorder"[Mesh:NoExp] OR "Depressive Disorder, Major"[Mesh] OR "Depressive Disorder, Treatment-Resistant"[Mesh] OR "Dysthymic Disorder"[Mesh] OR "Cognitive Dysfunction"[Mesh:NoExp] OR "Phobic Disorders"[Mesh] OR "Panic Disorder"[Mesh] OR "Cyclothymic Disorder"[Mesh] OR "Suicidal Ideation"[Mesh] OR "Sex Offenses"[Mesh] OR "Physical Abuse"[Mesh]) |
| **Racial and Ethnic Groups** | AND (“racially marginalized”[tiab] OR “marginalized population*”[tiab] OR “marginalized group*”[tiab] OR “disadvantaged population*”[tiab] OR “disadvantaged group*”[tiab] OR “vulnerable population*”[tiab] OR “vulnerable group*”[tiab] OR “African American*”[tiab] OR black[tiab] OR Latina[tiab] OR Latinas[tiab] OR Latino*[tiab] OR latinx[tiab] OR chicano*[tiab] OR chicana*[tiab] OR “Mexican American*”[tiab] OR Hispanic*[tiab] OR “Puerto Rican*”[tiab] OR biracial[tiab] OR “bi-racial”[tiab] OR “bi racial”[tiab] OR “mixed race”[tiab] OR indigenous[tiab] OR “Native American*”[tiab] OR “American Indian*”[tiab] OR “native Alaskan*”[tiab] OR “Alaska native*”[tiab] OR “Alaskan native*”[tiab] OR “Asian American*”[tiab] OR “Native Hawaiian*”[tiab] OR underserved[tiab] OR disparit*[tiab] OR inequit*[tiab] OR “low socioeconomic status”[tiab] OR “low socio economic status”[tiab] OR “low economic status”[tiab] OR “socioeconomically vulnerable”[tiab] OR “low SES”[tiab] OR “low income”[tiab] OR poverty[tiab] OR poor[tiab] OR minority[tiab] OR minorities[tiab] OR "African Americans"[Mesh] OR "Asian Americans"[Mesh] OR "Indigenous Peoples"[Mesh] OR "Indians, North American"[Mesh] OR "Native Hawaiian or Other Pacific Islander"[Mesh] OR "Mexican Americans"[Mesh] OR "Hispanic or Latino"[Mesh] OR "Health Disparity, Minority and Vulnerable Populations"[Mesh] OR "Poverty"[Mesh] OR "Ethnicity"[Mesh:NoExp] OR "Racial Groups"[Mesh:NoExp] OR "Socioeconomic Factors"[Mesh] OR "Economic Factors"[Mesh] OR "Economic Status"[Mesh] OR “women of color”[tiab] OR “woman of color”[tiab] OR multiethnic[tiab] OR “multi ethnic”[tiab] OR “diverse population*”[tiab] OR “diverse group*”[tiab] OR “ethnic diversity”[tiab] OR “racial diversity”[tiab] OR “racially diverse”[tiab] OR “ethnically diverse”[tiab] OR “marginalized women”[tiab] OR “BIPOC women”[tiab] OR “Chinese women”[tiab] OR “Japanese women”[tiab] OR “Latin American women”[tiab] OR “pacific islander women”[tiab] OR “cross cultural”[tiab] OR crosscultural[tiab] OR "Spanish speak*"[tiab] OR “latinoamerican*”[tiab]) |
| **United States** | AND (((ALABAMA[tiab] OR ALASKA[tiab] OR ARIZONA[tiab] OR ARKANSAS[tiab] OR CALIFORNIA[tiab] OR COLORADO[tiab] OR CONNECTICUT[tiab] OR DELAWARE[tiab] OR FLORIDA[tiab] OR GEORGIA[tiab] OR HAWAII[tiab] OR IDAHO[tiab] OR ILLINOIS[tiab] OR INDIANA[tiab] OR IOWA[tiab] OR KANSAS[tiab] OR KENTUCKY[tiab] OR LOUISIANA[tiab] OR MAINE[tiab] OR MARYLAND[tiab] OR MASSACHUSETTS[tiab] OR MICHIGAN[tiab] OR MINNESOTA[tiab] OR MISSISSIPPI[tiab] OR MISSOURI[tiab] OR MONTANA[tiab] OR NEBRASKA[tiab] OR NEVADA[tiab] OR "NEW HAMPSHIRE"[tiab] OR "NEW JERSEY"[tiab] OR "NEW MEXICO"[tiab] OR "NEW YORK"[tiab] OR "NORTH CAROLINA"[tiab] OR "NORTH DAKOTA"[tiab] OR OHIO[tiab] OR OKLAHOMA[tiab] OR OREGON[tiab] OR PENNSYLVANIA[tiab] OR "RHODE ISLAND"[tiab] OR "SOUTH CAROLINA"[tiab] OR "SOUTH DAKOTA"[tiab] OR TENNESSEE[tiab] OR TEXAS[tiab] OR UTAH[tiab] OR VERMONT[tiab] OR VIRGINIA[tiab] OR WASHINGTON[tiab] OR "WEST VIRGINIA"[tiab] OR WISCONSIN[tiab] OR WYOMING[tiab] OR USA[tiab] OR "United States"[tiab] OR US[ti] OR u.s.[ti] OR u.s.a.[tiab] OR American*[tiab] OR GUAM[tiab] OR "PUERTO RICO"[tiab] OR "VIRGIN ISLANDS"[tiab] OR "American Samoa"[tiab] OR "Northern Mariana Islands"[tiab] OR AL[tiab] OR AK[tiab] OR AZ[tiab] OR AR[tiab] OR CA[tiab] OR CO[tiab] OR CT[tiab] OR DE[tiab] OR FL[tiab] OR GA[tiab] OR HI[tiab] OR ID[tiab] OR IL[tiab] OR IA[tiab] OR KS[tiab] OR KY[tiab] OR LA[tiab] OR ME[tiab] OR MD[tiab] OR MA[tiab] OR MI[tiab] OR MN[tiab] OR MS[tiab] OR MO[tiab] OR MT[tiab] OR NE[tiab] OR NV[tiab] OR NH[tiab] OR NJ[tiab] OR NM[tiab] OR NY[tiab] OR NC[tiab] OR ND[tiab] OR OH[tiab] OR OK[tiab] OR PA[tiab] OR RI[tiab] OR SC[tiab] OR SD[tiab] OR TN[tiab] OR TX[tiab] OR UT[tiab] OR VT[tiab] OR VA[tiab] OR WA[tiab] OR WV[tiab] OR WI[tiab] OR WY[tiab] OR "New York City"[tiab] OR "Los Angeles"[tiab] OR Houston[tiab] OR Dallas[tiab] OR Miami[tiab] OR Chicago[tiab] OR Dallas[tiab] OR Detroit[tiab] OR Seattle[tiab] OR "San Francisco"[tiab] OR Sacramento[tiab] OR "San Diego"[tiab] OR Boston[tiab] OR "Washington DC"[tiab] OR “district of columbia”[tiab] OR Baltimore[tiab] OR Pittsburgh[tiab] OR Philadelphia[tiab] OR Denver[tiab] OR Phoenix[tiab] OR "New Orleans"[tiab] OR ALABAMA[mesh] OR ALASKA[mesh] OR ARIZONA[mesh] OR ARKANSAS[mesh] OR CALIFORNIA[mesh] OR COLORADO[mesh] OR CONNECTICUT[mesh] OR DELAWARE[mesh] OR FLORIDA[mesh] OR GEORGIA[mesh] OR HAWAII[mesh] OR IDAHO[mesh] OR ILLINOIS[mesh] OR INDIANA[mesh] OR IOWA[mesh] OR KANSAS[mesh] OR KENTUCKY[mesh] OR LOUISIANA[mesh] OR MAINE[mesh] OR MARYLAND[mesh] OR MASSACHUSETTS[mesh] OR MICHIGAN[mesh] OR MINNESOTA[mesh] OR MISSISSIPPI[mesh] OR MISSOURI[mesh] OR MONTANA[mesh] OR NEBRASKA[mesh] OR NEVADA[mesh] OR "NEW HAMPSHIRE"[mesh] OR "NEW JERSEY"[mesh] OR "NEW MEXICO"[mesh] OR "NEW YORK"[mesh] OR "NORTH CAROLINA"[mesh] OR "NORTH DAKOTA"[mesh] OR OHIO[mesh] OR OKLAHOMA[mesh] OR OREGON[mesh] OR PENNSYLVANIA[mesh] OR "RHODE ISLAND"[mesh] OR "SOUTH CAROLINA"[mesh] OR "SOUTH DAKOTA"[mesh] OR TENNESSEE[mesh] OR TEXAS[mesh] OR UTAH[mesh] OR VERMONT[mesh] OR VIRGINIA[mesh] OR WASHINGTON[mesh] OR "WEST VIRGINIA"[mesh] OR WISCONSIN[mesh] OR WYOMING[mesh] OR "United States"[mesh] OR "American Samoa"[Mesh] OR GUAM[mesh] OR "PUERTO RICO"[mesh] OR "United States VIRGIN ISLANDS"[mesh] OR "New York City"[Mesh] OR "District of Columbia"[Mesh] OR "Philadelphia"[Mesh] OR "Baltimore"[Mesh] OR Boston[mesh] OR Chicago[mesh] OR "Los Angeles"[mesh] OR "New Orleans"[mesh] OR "San Francisco"[mesh] OR Appalachian Region[mesh] OR Great Lakes Region[mesh] OR Mid-Atlantic Region[mesh] OR Midwestern United States[mesh] OR New England[mesh] OR Northwestern United States[mesh] OR Pacific States[mesh] OR Southeastern United States[mesh] OR Southwestern United States[mesh]) NOT dollar*[tiab]) OR ((Northwest*[tiab] OR northeast*[tiab] OR southwest*[tiab] OR southeast*[tiab] OR midwest*[tiab] OR southern[tiab] OR northern[tiab] OR "new england"[tiab] OR "mid atlantic"[tiab] OR "Great lakes region"[tiab] OR Appalachia*[tiab] OR “pacific northwest”[tiab]) AND (USA[tiab] OR "United States"[tiab] OR US[ti] OR u.s.[ti] OR u.s.a.[tiab]))) |
|  |  |
| **Limits Applied:** | Language: English[lang] |
|  | Publication year: ("2005/01/01"[Date - Publication] : "2021/12/31"[Date - Publication]) |
|  | NOT (letter[ptyp] OR editorial[ptyp] OR comment[ptyp] OR news[ptyp] OR "Congress"[Publication Type] OR "Consensus Development Conference"[Publication Type] OR editorial[tiab] OR commentary[tiab] OR “conference abstract*”[tiab] OR “conference proceeding*”[tiab] OR "Published Erratum"[Publication Type] OR errata[tiab] OR erratum[tiab] OR corrigenda[tiab] OR corrigendum[tiab] OR protocol[ti] OR protocols[ti]) |
|  | NOT ("Animals"[Mesh] NOT ("Animals"[Mesh] AND "Humans"[Mesh]))  NOT (mice[tiab] OR mouse[tiab] OR rat[tiab] OR rats[tiab] OR rodent*[tiab] OR murine*[tiab] OR dog[tiab] OR dogs[tiab] OR pig[tiab] OR pigs[tiab] OR piglet*[tiab] OR swine[tiab] OR porcine*[tiab] OR sheep[tiab] OR animal*[tiab] OR Dogs[mesh] OR Swine[mesh] OR Rodentia[mesh] OR Models, Animal[mesh] OR Animal Experimentation[mesh]) |

**Database:** Embase

**Vendor:** Elsevier

**Date of search:** February 8, 2022

**Limits:** Source: Embase; Publication Year: 2005–2021; Language: English; Document Type: exclude letter, comment, editorial, retracted publication, meeting abstract, conference abstract. Species: exclude animal studies.

| **Concept** | **Search Terms Used** |
| --- | --- |
| **Menopause** | ('menopause'/de OR 'premenopause'/exp OR menopause:ti,ab OR menopausal:ti,ab OR 'peri-menopaus*':ti,ab OR perimenopaus*:ti,ab) |
| **Mental Health Conditions** | AND ('mental health'/de OR 'psychological well-being'/de OR 'anxiety disorder'/exp OR 'mood disorder'/de OR 'depression'/de OR 'major depression'/exp OR 'treatment resistant depression'/exp OR 'major affective disorder'/exp OR 'psychosis'/exp OR 'schizoaffective psychosis'/exp OR 'disruptive mood dysregulation disorder'/exp OR 'schizophrenia'/exp OR 'bipolar disorder'/exp OR 'phobia'/exp OR 'borderline state'/exp OR 'domestic violence'/exp OR 'posttraumatic stress disorder'/de OR 'premenstrual dysphoric disorder'/exp OR 'suicidal ideation'/exp OR 'cognitive defect'/de OR 'mild cognitive impairment'/de OR 'panic disorder*':ti,ab OR 'social phobia*':ti,ab OR 'mental illness*':ti,ab OR 'mental disorder*':ti,ab OR 'mental health':ti,ab OR 'mentally ill':ti,ab OR 'mood disorder*':ti,ab OR 'affective disorder*':ti,ab OR depression:ti,ab OR depressed:ti,ab OR 'depressive disorder*':ti,ab OR psychosis:ti,ab OR psychoses:ti,ab OR 'psychotic disorder*':ti,ab OR 'schizoaffective disorder*':ti,ab OR anxiety:ti,ab OR phobia*:ti,ab OR schizophren*:ti,ab OR bipolar:ti,ab OR 'manic disorder*':ti,ab OR 'manic depressive':ti,ab OR 'obsessive compulsive disorder*':ti,ab OR 'disruptive mood dysregulation disorder*':ti,ab OR 'borderline personality disorder*':ti,ab OR 'panic disorder*':ti,ab OR 'phobic disorder*':ti,ab OR 'Dysthymic Disorder*':ti,ab OR 'Cyclothymic Disorder*':ti,ab OR 'family violence':ti,ab OR 'domestic violence':ti,ab OR 'spousal abuse':ti,ab OR 'spouse abuse':ti,ab OR 'domestic abuse':ti,ab OR 'elder abuse':ti,ab OR 'child abuse':ti,ab OR 'partner violence':ti,ab OR 'battered woman':ti,ab OR 'battered women':ti,ab OR PTSD:ti,ab OR 'post traumatic stress disorder*':ti,ab OR 'posttraumatic stress disorder*':ti,ab OR 'premenstrual dysphoric disorder*':ti,ab OR 'premenstrual dysphoric syndrome*':ti,ab OR 'pre-menstrual dysphoric disorder*':ti,ab OR 'co occurring substance use':ti,ab OR 'cooccurring substance use':ti,ab OR 'cognitive decline*':ti,ab OR 'cognitive dysfunction*':ti,ab OR 'cognitive impairment*':ti,ab OR 'Mild Neurocognitive Disorder*':ti,ab OR 'suicidal ideation*':ti,ab OR 'suicidal thought*':ti,ab OR 'Suicide ideation*':ti,ab OR 'suicidal behavior*':ti,ab OR 'suicide ideation*':ti,ab OR 'sexual abuse'/exp OR 'physical violence'/exp OR 'sexual abuse':ti,ab OR 'physical abuse':ti,ab OR 'physical violence':ti,ab) |
| **Racial and Ethnic Groups** | AND ('racially marginalized':ti,ab OR 'marginalized population*':ti,ab OR 'marginalized group*':ti,ab OR 'disadvantaged population*':ti,ab OR 'disadvantaged group*':ti,ab OR 'vulnerable population*':ti,ab OR 'vulnerable group*':ti,ab OR 'African American*':ti,ab OR black:ti,ab OR Latina:ti,ab OR Latinas:ti,ab OR Latino*:ti,ab OR latinx:ti,ab OR chicano*:ti,ab OR chicana*:ti,ab OR 'Mexican American*':ti,ab OR Hispanic*:ti,ab OR 'Puerto Rican*':ti,ab OR biracial:ti,ab OR 'bi-racial':ti,ab OR 'bi racial':ti,ab OR 'mixed race':ti,ab OR indigenous:ti,ab OR 'Native American*':ti,ab OR 'American Indian*':ti,ab OR 'native Alaskan*':ti,ab OR 'Alaska native*':ti,ab OR 'Alaskan native*':ti,ab OR 'Asian American*':ti,ab OR 'Native Hawaiian*':ti,ab OR underserved:ti,ab OR disparit*:ti,ab OR inequit*:ti,ab OR poverty:ti,ab OR poor:ti,ab OR 'low socioeconomic status':ti,ab OR 'low socio economic status':ti,ab OR 'low economic status':ti,ab OR 'socioeconomically vulnerable':ti,ab OR 'low SES':ti,ab OR 'low income':ti,ab OR minority:ti,ab OR minorities:ti,ab OR 'African American'/exp OR 'Asian American'/exp OR 'Hispanic'/exp OR 'Alaska Native'/exp OR 'American Indian'/exp OR 'ancestry group'/de OR 'poverty'/exp OR 'vulnerable population'/exp OR 'disadvantaged population'/exp OR 'ethnic group'/de OR 'socioeconomics'/de OR 'household economic status'/exp OR 'socioeconomic distribution'/exp OR 'socioeconomic vulnerability'/exp OR 'women of color':ti,ab OR 'woman of color':ti,ab OR multiethnic:ti,ab OR 'multi ethnic':ti,ab OR 'diverse population*':ti,ab OR 'diverse group*':ti,ab OR 'ethnic diversity':ti,ab OR 'racial diversity':ti,ab OR 'racially diverse':ti,ab OR 'ethnically diverse':ti,ab OR 'marginalized women':ti,ab OR 'BIPOC women':ti,ab OR 'chinese women':ti,ab OR 'Japanese women':ti,ab OR 'Latin American women':ti,ab OR 'pacific islander women':ti,ab OR 'cross cultural':ti,ab OR crosscultural:ti,ab OR 'Spanish speak*':ti,ab OR 'latinoamerican*':ti,ab) |
| **United States** | AND (((ALABAMA:ti,ab OR ALASKA:ti,ab OR ARIZONA:ti,ab OR ARKANSAS:ti,ab OR CALIFORNIA:ti,ab OR COLORADO:ti,ab OR CONNECTICUT:ti,ab OR DELAWARE:ti,ab OR FLORIDA:ti,ab OR GEORGIA:ti,ab OR HAWAII:ti,ab OR IDAHO:ti,ab OR ILLINOIS:ti,ab OR INDIANA:ti,ab OR IOWA:ti,ab OR KANSAS:ti,ab OR KENTUCKY:ti,ab OR LOUISIANA:ti,ab OR MAINE:ti,ab OR MARYLAND:ti,ab OR MASSACHUSETTS:ti,ab OR MICHIGAN:ti,ab OR MINNESOTA:ti,ab OR MISSISSIPPI:ti,ab OR MISSOURI:ti,ab OR MONTANA:ti,ab OR NEBRASKA:ti,ab OR NEVADA:ti,ab OR 'NEW HAMPSHIRE':ti,ab OR 'NEW JERSEY':ti,ab OR 'NEW MEXICO':ti,ab OR 'NEW YORK':ti,ab OR 'NORTH CAROLINA':ti,ab OR 'NORTH DAKOTA':ti,ab OR OHIO:ti,ab OR OKLAHOMA:ti,ab OR OREGON:ti,ab OR PENNSYLVANIA:ti,ab OR 'RHODE ISLAND':ti,ab OR 'SOUTH CAROLINA':ti,ab OR 'SOUTH DAKOTA':ti,ab OR TENNESSEE:ti,ab OR TEXAS:ti,ab OR UTAH:ti,ab OR VERMONT:ti,ab OR VIRGINIA:ti,ab OR WASHINGTON:ti,ab OR 'WEST VIRGINIA':ti,ab OR WISCONSIN:ti,ab OR WYOMING:ti,ab OR USA:ti,ab OR 'United States':ti,ab OR US:ti OR u.s.:ti OR u.s.a.:ti,ab OR American*:ti,ab OR GUAM:ti,ab OR 'PUERTO RICO':ti,ab OR 'VIRGIN ISLANDS':ti,ab OR 'American Samoa':ti,ab OR 'Northern Mariana Islands':ti,ab OR AL:ti,ab OR AK:ti,ab OR AZ:ti,ab OR AR:ti,ab OR CA:ti,ab OR CO:ti,ab OR CT:ti,ab OR DE:ti,ab OR FL:ti,ab OR GA:ti,ab OR HI:ti,ab OR ID:ti,ab OR IL:ti,ab OR IA:ti,ab OR KS:ti,ab OR KY:ti,ab OR LA:ti,ab OR ME:ti,ab OR MD:ti,ab OR MA:ti,ab OR MI:ti,ab OR MN:ti,ab OR MS:ti,ab OR MO:ti,ab OR MT:ti,ab OR NE:ti,ab OR NV:ti,ab OR NH:ti,ab OR NJ:ti,ab OR NM:ti,ab OR NY:ti,ab OR NC:ti,ab OR ND:ti,ab OR OH:ti,ab OR OK:ti,ab OR PA:ti,ab OR RI:ti,ab OR SC:ti,ab OR SD:ti,ab OR TN:ti,ab OR TX:ti,ab OR UT:ti,ab OR VT:ti,ab OR VA:ti,ab OR WA:ti,ab OR WV:ti,ab OR WI:ti,ab OR WY:ti,ab OR 'New York City':ti,ab OR 'Los Angeles':ti,ab OR Houston:ti,ab OR Dallas:ti,ab OR Miami:ti,ab OR Chicago:ti,ab OR Dallas:ti,ab OR Detroit:ti,ab OR Seattle:ti,ab OR 'San Francisco':ti,ab OR Sacramento:ti,ab OR 'San Diego':ti,ab OR Boston:ti,ab OR 'Washington DC':ti,ab OR 'district of Columbia':ti,ab OR Baltimore:ti,ab OR Pittsburgh:ti,ab OR Philadelphia:ti,ab OR Denver:ti,ab OR Phoenix:ti,ab OR 'New Orleans':ti,ab OR ALABAMA/de OR ALASKA/de OR ARIZONA/de OR ARKANSAS/de OR CALIFORNIA/de OR COLORADO/de OR CONNECTICUT/de OR DELAWARE/de OR FLORIDA/de OR 'District of Columbia'/exp OR 'Georgia (U.S.)'/exp OR HAWAII/de OR IDAHO/de OR ILLINOIS/de OR INDIANA/de OR IOWA/de OR KANSAS/de OR KENTUCKY/de OR LOUISIANA/de OR MAINE/de OR MARYLAND/de OR MASSACHUSETTS/de OR MICHIGAN/de OR MINNESOTA/de OR MISSISSIPPI/de OR MISSOURI/de OR MONTANA/de OR NEBRASKA/de OR NEVADA/de OR 'NEW HAMPSHIRE'/de OR 'NEW JERSEY'/de OR 'NEW MEXICO'/de OR 'NEW YORK'/de OR 'NORTH CAROLINA'/de OR 'NORTH DAKOTA'/de OR OHIO/de OR OKLAHOMA/de OR OREGON/de OR PENNSYLVANIA/de OR 'RHODE ISLAND'/de OR 'SOUTH CAROLINA'/de OR 'SOUTH DAKOTA'/de OR TENNESSEE/de OR TEXAS/de OR UTAH/de OR VERMONT/de OR VIRGINIA/de OR WASHINGTON/de OR 'WEST VIRGINIA'/de OR WISCONSIN/de OR WYOMING/de OR 'United States'/exp OR 'American Samoa'/exp OR 'Guam'/exp OR 'Puerto Rico'/exp OR 'Virgin Islands (U.S.)'/exp) NOT dollar*:ti,ab) OR ((Northwest*:ti,ab OR northeast*:ti,ab OR southwest*:ti,ab OR southeast*:ti,ab OR midwest*:ti,ab OR southern:ti,ab OR northern:ti,ab OR 'new england':ti,ab OR 'mid atlantic':ti,ab OR 'Great lakes region':ti,ab OR Appalachia*:ti,ab OR 'pacific northwest':ti,ab) AND (USA:ti,ab OR 'United States':ti,ab OR US:ti OR u.s.:ti OR u.s.a.:ti,ab))) |
|  |  |
| **Limits Applied** | Language: AND [english]/lim |
|  | Source: AND (([embase]/lim OR [embase classic]/lim) |
|  | Publication date: AND [2005-2021]/py) |
|  | NOT ([conference abstract]/lim OR [conference paper]/lim OR [conference review]/lim OR [data papers]/lim OR [editorial]/lim OR [erratum]/lim OR [letter]/lim OR [note]/lim OR [review]/lim OR [short survey]/lim OR 'conference abstract'/exp OR 'conference paper'/exp OR 'data paper'/exp OR 'editorial'/exp OR 'letter'/exp OR 'erratum'/exp OR 'note'/exp OR 'short survey'/exp OR corrigenda:ti,ab OR corrigendum:ti,ab OR erratum:ti,ab OR errata:ti,ab OR “conference abstract*”:ti,ab OR “conference proceeding*”:ti,ab OR letter:ti,ab OR editorial:ti,ab OR commentary:ti,ab) |
|  | NOT ([animals]/lim NOT ([animals]/lim AND [humans]/lim))  NOT (mice:ti,ab OR mouse:ti,ab OR rat:ti,ab OR rats:ti,ab OR dog:ti,ab OR dogs:ti,ab OR pig:ti,ab OR pigs:ti,ab OR piglet:ti,ab OR piglets:ti,ab OR swine:ti,ab OR sheep:ti,ab OR porcine*:ti,ab OR rodent*:ti,ab OR animal*:ti,ab OR 'dog'/exp OR 'pig'/exp OR 'rodent'/exp OR [animal cell]/lim OR [animal experiment]/lim OR [animal model]/lim OR [animal tissue]/lim) |

**Database:** Web of Science: Core Collection

**Vendor:** Clarivate Analytics

**Date of search:** February 17, 2022

**Limits:** Language: English; Publication Year: 2005–2021; Field: Topic field (title, abstract, keywords, and Keywords Plus); Document Type: exclude letter, editorial, comment, conference abstract, meeting abstract, meeting summary, proceedings paper. Species: exclude animal studies.

| **Concept** | **Search Set** | **Search Terms Used** |
| --- | --- | --- |
| **Menopause** | **#1** | TS=((menopause OR menopausal OR “peri-menopaus*” OR perimenopaus*)) |
| **Mental Health Conditions** | **#2** | TS=( (“mental illness*” OR “mental disorder*” OR “mental health” OR “mentally ill” OR “mood disorder*” OR “affective disorder*” OR depression OR depressed OR “depressive disorder*” OR psychosis OR psychoses OR “psychotic disorder*” OR “schizoaffective disorder*” OR anxiety OR “panic disorder*” OR phobia* OR “phobic disorder*” OR schizophren* OR bipolar OR “manic disorder*” OR “manic depressive” OR “obsessive compulsive disorder*” OR “disruptive mood dysregulation disorder*” OR “borderline personality disorder*” OR “Dysthymic Disorder*” OR “Cyclothymic Disorder*” OR “family violence” OR “domestic violence” OR “spousal abuse” OR “spouse abuse” OR “domestic abuse” OR “elder abuse” OR “child abuse” OR “partner violence” OR “battered woman” OR “battered women” OR PTSD OR “post traumatic stress disorder*” OR “posttraumatic stress disorder*” OR “premenstrual dysphoric disorder*” OR “premenstrual dysphoric syndrome*” OR “pre-menstrual dysphoric disorder*” OR “co occurring substance use” OR “cooccurring substance use” OR “cognitive decline*” OR “cognitive dysfunction*” OR “cognitive impairment*” OR “Mild Neurocognitive Disorder*” OR “suicidal ideation*” OR “suicidal thought*” OR “suicidal behavior*” OR “suicide ideation*” OR “sexual abuse” OR “physical abuse” OR “physical violence”)) |
| **United States** | **#3** | TS=( (((ALABAMA OR ALASKA OR ARIZONA OR ARKANSAS OR CALIFORNIA OR COLORADO OR CONNECTICUT OR DELAWARE OR FLORIDA OR GEORGIA OR HAWAII OR IDAHO OR ILLINOIS OR INDIANA OR IOWA OR KANSAS OR KENTUCKY OR LOUISIANA OR MAINE OR MARYLAND OR MASSACHUSETTS OR MICHIGAN OR MINNESOTA OR MISSISSIPPI OR MISSOURI OR MONTANA OR NEBRASKA OR NEVADA OR "NEW HAMPSHIRE" OR "NEW JERSEY" OR "NEW MEXICO" OR "NEW YORK" OR "NORTH CAROLINA" OR "NORTH DAKOTA" OR OHIO OR OKLAHOMA OR OREGON OR PENNSYLVANIA OR "RHODE ISLAND" OR "SOUTH CAROLINA" OR "SOUTH DAKOTA" OR TENNESSEE OR TEXAS OR UTAH OR VERMONT OR VIRGINIA OR WASHINGTON OR "WEST VIRGINIA" OR WISCONSIN OR WYOMING OR USA OR "United States" OR US OR u.s. OR u.s.a. OR American* OR GUAM OR "PUERTO RICO" OR "VIRGIN ISLANDS" OR "American Samoa" OR "Northern Mariana Islands" OR "New York City" OR "Los Angeles" OR Houston OR Dallas OR Miami OR Chicago OR Dallas OR Detroit OR Seattle OR "San Francisco" OR Sacramento OR "San Diego" OR Boston OR "Washington DC" OR “district of columbia” OR Baltimore OR Pittsburgh OR Philadelphia OR Denver OR Phoenix OR "New Orleans") NOT dollar*) OR ((Northwest* OR northeast* OR southwest* OR southeast* OR midwest* OR southern OR northern OR "new england" OR "mid atlantic" OR "Great lakes region" OR Appalachia* OR “pacific northwest”) AND (USA OR "United States" OR US OR u.s. OR u.s.a.)))) |
| **Racial and Ethnic Groups** | **#4** | TS=((“racially marginalized” OR “marginalized population*” OR “marginalized group*” OR “disadvantaged population*” OR “disadvantaged group*” OR “vulnerable population*” OR “vulnerable group*” OR “African American*” OR black OR Latina OR Latinas OR Latino* OR latinx OR chicano* OR chicana* OR “Mexican American*” OR Hispanic* OR “Puerto Rican*” OR biracial OR “bi-racial” OR “bi racial” OR “mixed race” OR indigenous OR “Native American*” OR “American Indian*” OR “native Alaskan*” OR “Alaska native*” OR “Alaskan native*” OR “Asian American*” OR “Native Hawaiian*” OR underserved OR disparit* OR inequit* OR poverty OR poor OR “low socioeconomic status” OR “low economic status” OR “low socio economic status” OR “low SES” OR “low income” OR minority OR minorities OR “socioeconomically vulnerable” OR “women of color” OR “woman of color” OR multiethnic OR “multi ethnic” OR “diverse population*” OR “diverse group*” OR “ethnic diversity” OR “racial diversity” OR “racially diverse” OR “ethnically diverse” OR “marginalized women” OR “BIPOC women” OR “chinese women” OR “Japanese women” OR “latin American women” OR “pacific islander women” OR “cross cultural” OR crosscultural OR “Spanish speak*” OR latinoamerican*)) |
|  | **#5** | #1 AND #2 AND #3 AND #4 |
|  | **#6** | #5 AND English AND (2005–2021) |
|  | **#7** | #6 NOT TS=(mice OR mouse OR rat OR rats OR rodent* OR murine* OR dog OR dogs OR pig OR pigs OR piglet* OR swine OR porcine* OR sheep OR animal*) |
|  | **#8** | #7 NOT TS=(corrigenda OR corrigendum OR erratum OR errata OR “conference abstract” OR “conference abstracts” OR “conference proceeding” OR “conference proceedings” OR editorial OR commentary OR protocol OR protocols) |
|  | **#9** | #8 AND Document Type: Exclude all but Articles, Review Articles, Early Access |
|  |  |  |
| **Limits Applied** |  | Language: English |
|  |  | Publication year: 2005–2021 |
|  |  | NOT TS=(corrigenda OR corrigendum OR erratum OR errata OR “conference abstract” OR “conference abstracts” OR “conference proceeding” OR “conference proceedings” OR editorial OR commentary OR protocol OR protocols) |
|  |  | NOT TS=(mice OR mouse OR rat OR rats OR rodent* OR murine* OR dog OR dogs OR pig OR pigs OR piglet* OR swine OR porcine* OR sheep OR animal*) |

**Notes:** Web of Science: Core Collection includes the following databases available through our library subscription: Science Citation Index Expanded (SCI-EXPANDED)--1900-present; Social Sciences Citation Index (SSCI)--1900-present; Conference Proceedings Citation Index – Science (CPCI-S)--1990-present; Conference Proceedings Citation Index – Social Science & Humanities (CPCI-SSH)--1990-present; Book Citation Index – Science (BKCI-S)--2005-present; Book Citation Index – Social Sciences & Humanities (BKCI-SSH)--2005-present; Emerging Sources Citation Index (ESCI)--2005-present; Current Chemical Reactions (CCR-EXPANDED)--1985-present.

**Database:** CINAHL Plus

**Vendor:** Ebscohost

**Date of search:** February 17, 2022

**Limits:** Publication Year: 2005–2021; Language: English; Field: title and abstract fields search; Expanders: Apply equivalent subjects; Publication Type: exclude case study, doctoral dissertation, conference abstract, conference proceedings, letter, editorial, comment. Species: exclude animal studies.

| **Concept** | **Search Set** | **Search Terms Used** |
| --- | --- | --- |
|  | **#1** | Title: (menopause OR menopausal OR “peri-menopaus*” OR perimenopaus*) |
| **Menopause** | **#2** | Abstract: (menopause OR menopausal OR “peri-menopaus*” OR perimenopaus*) |
|  | **#3** | Exact Subject Headings: (MH "Menopause") OR (MH "Perimenopause") OR (MH "Menopause, Premature") |
|  | **#4** | #1 OR #2 OR #3 |
|  | **#5** | Title: (“mental illness*” OR “mental disorder*” OR “mental health” OR “mentally ill” OR “mood disorder*” OR “affective disorder*” OR depression OR depressed OR “depressive disorder*” OR psychosis OR psychoses OR “psychotic disorder*” OR “schizoaffective disorder*” OR anxiety OR “panic disorder*” OR phobia* OR “phobic disorder*” OR schizophren* OR bipolar OR “manic disorder*” OR “manic depressive” OR “obsessive compulsive disorder*” OR “disruptive mood dysregulation disorder*” OR “borderline personality disorder*” OR “Dysthymic Disorder*” OR “Cyclothymic Disorder*” OR “family violence” OR “domestic violence” OR “spousal abuse” OR “spouse abuse” OR “domestic abuse” OR “elder abuse” OR “child abuse” OR “partner violence” OR “battered woman” OR “battered women” OR PTSD OR “post traumatic stress disorder*” OR “posttraumatic stress disorder*” OR “premenstrual dysphoric disorder*” OR “premenstrual dysphoric syndrome*” OR “pre-menstrual dysphoric disorder*” OR “co occurring substance use” OR “cooccurring substance use” OR “cognitive decline*” OR “cognitive dysfunction*” OR “cognitive impairment*” OR “Mild Neurocognitive Disorder*” OR “suicidal ideation*” OR “suicidal thought*” OR “suicidal behavior*” OR “suicide ideation*” OR “sexual abuse” OR “physical abuse” OR “physical violence”) |
| **Mental Health Conditions** | **#6** | Abstract: (“mental illness*” OR “mental disorder*” OR “mental health” OR “mentally ill” OR “mood disorder*” OR “affective disorder*” OR depression OR depressed OR “depressive disorder*” OR psychosis OR psychoses OR “psychotic disorder*” OR “schizoaffective disorder*” OR anxiety OR “panic disorder*” OR phobia* OR “phobic disorder*” OR schizophren* OR bipolar OR “manic disorder*” OR “manic depressive” OR “obsessive compulsive disorder*” OR “disruptive mood dysregulation disorder*” OR “borderline personality disorder*” OR “Dysthymic Disorder*” OR “Cyclothymic Disorder*” OR “family violence” OR “domestic violence” OR “spousal abuse” OR “spouse abuse” OR “domestic abuse” OR “elder abuse” OR “child abuse” OR “partner violence” OR “battered woman” OR “battered women” OR PTSD OR “post traumatic stress disorder*” OR “posttraumatic stress disorder*” OR “premenstrual dysphoric disorder*” OR “premenstrual dysphoric syndrome*” OR “pre-menstrual dysphoric disorder*” OR “co occurring substance use” OR “cooccurring substance use” OR “cognitive decline*” OR “cognitive dysfunction*” OR “cognitive impairment*” OR “Mild Neurocognitive Disorder*” OR “suicidal ideation*” OR “suicidal thought*” OR “suicidal behavior*” OR “suicide ideation*” OR “sexual abuse” OR “physical abuse” OR “physical violence”) |
|  | **#7** | Exact Subject Heading: ((MH "Mental Health") OR (MM "Mental Disorders") OR (MH "Stress Disorders, Post-Traumatic+") OR (MH "Affective Disorders") OR (MH "Premenstrual Dysphoric Disorder") OR (MH "Depression+") OR (MH "Bipolar Disorder+") OR (MH "Anxiety Disorders+") OR (MH "Panic Disorder") OR (MH "Phobic Disorders+") OR (MH "Schizophrenia") OR (MH "Psychotic Disorders") OR (MH "Affective Disorders, Psychotic") OR (MH "Schizoaffective Disorder") OR (MH "Borderline Personality Disorder") OR (MH "Domestic Violence+") OR (MH "Intimate Partner Violence") OR (MH "Elder Abuse") OR (MH "Child Abuse") OR (MH "Battered Women") OR (MH "Dysthymic Disorder") OR (MH "Cyclothymic Disorder") OR (MH "Suicidal Ideation") OR (MH "Sexual Abuse+")) |
|  | **#8** | #5 OR #6 OR #7 |
|  | **#9** | Exact Subject Heading: (MH "United States+") OR (MH "United States by Individual State+") OR (MH "United States by Region") OR (MH "Appalachian Region+") OR (MH "Great Lakes Region") OR (MH "Mid Atlantic Region") OR (MH "Midwestern United States") OR (MH "New England") OR (MH "Northwestern United States") OR (MH "Southeastern United States") OR (MH "Southwestern United States") OR (MH "Alabama") OR (MH "Alaska") OR (MH "Arizona") OR (MH "Arkansas") OR (MH "California") OR (MH "Connecticut") OR (MH "Delaware") OR (MH "Colorado") OR (MH "District of Columbia") OR (MH "Florida") OR (MH "Georgia") OR (MH "Hawaii") OR (MH "Idaho") OR (MH "Illinois") OR (MH "Indiana") OR (MH "Iowa") OR (MH "Kansas") OR (MH "Kentucky") OR (MH "Louisiana") OR (MH "Maine") OR (MH "Maryland") OR (MH "Massachusetts") OR (MH "Michigan") OR (MH "Mississippi") OR (MH "Minnesota") OR (MH "Missouri") OR (MH "Montana") OR (MH "Nebraska") OR (MH "Nevada") OR (MH "New Hampshire") OR (MH "New Jersey") OR (MH "New Mexico") OR (MH "New York") OR (MH "North Carolina") OR (MH "North Dakota") OR (MH "Ohio") OR (MH "Oklahoma") OR (MH "Pennsylvania") OR (MH "Rhode Island") OR (MH "South Carolina") OR (MH "South Dakota") OR (MH "Tennessee") OR (MH "Texas") OR (MH "Utah") OR (MH "Vermont") OR (MH "Virginia") OR (MH "West Virginia") OR (MH "Washington") OR (MH "Wisconsin") OR (MH "Wyoming") OR (MH "Guam") OR (MH "American Samoa") OR (MH "Puerto Rico") OR (MH "Virgin Islands of the United States") |
| **United States** | **#10** | Title: (((ALABAMA OR ALASKA OR ARIZONA OR ARKANSAS OR CALIFORNIA OR COLORADO OR CONNECTICUT OR DELAWARE OR FLORIDA OR GEORGIA OR HAWAII OR IDAHO OR ILLINOIS OR INDIANA OR IOWA OR KANSAS OR KENTUCKY OR LOUISIANA OR MAINE OR MARYLAND OR MASSACHUSETTS OR MICHIGAN OR MINNESOTA OR MISSISSIPPI OR MISSOURI OR MONTANA OR NEBRASKA OR NEVADA OR "NEW HAMPSHIRE" OR "NEW JERSEY" OR "NEW MEXICO" OR "NEW YORK" OR "NORTH CAROLINA" OR "NORTH DAKOTA" OR OHIO OR OKLAHOMA OR OREGON OR PENNSYLVANIA OR "RHODE ISLAND" OR "SOUTH CAROLINA" OR "SOUTH DAKOTA" OR TENNESSEE OR TEXAS OR UTAH OR VERMONT OR VIRGINIA OR WASHINGTON OR "WEST VIRGINIA" OR WISCONSIN OR WYOMING OR USA OR "United States" OR US OR u.s. OR u.s.a. OR American* OR GUAM OR "PUERTO RICO" OR "VIRGIN ISLANDS" OR "American Samoa" OR "Northern Mariana Islands" OR "New York City" OR "Los Angeles" OR Houston OR Dallas OR Miami OR Chicago OR Dallas OR Detroit OR Seattle OR "San Francisco" OR Sacramento OR "San Diego" OR Boston OR "Washington DC" OR “district of columbia” OR Baltimore OR Pittsburgh OR Philadelphia OR Denver OR Phoenix OR "New Orleans") NOT dollar*) OR ((Northwest* OR northeast* OR southwest* OR southeast* OR midwest* OR southern OR northern OR "new england" OR "mid atlantic" OR "Great lakes region" OR Appalachia* OR “pacific northwest”) AND (USA OR "United States" OR US OR u.s. OR u.s.a.))) |
|  | **#11** | Abstract: (((ALABAMA OR ALASKA OR ARIZONA OR ARKANSAS OR CALIFORNIA OR COLORADO OR CONNECTICUT OR DELAWARE OR FLORIDA OR GEORGIA OR HAWAII OR IDAHO OR ILLINOIS OR INDIANA OR IOWA OR KANSAS OR KENTUCKY OR LOUISIANA OR MAINE OR MARYLAND OR MASSACHUSETTS OR MICHIGAN OR MINNESOTA OR MISSISSIPPI OR MISSOURI OR MONTANA OR NEBRASKA OR NEVADA OR "NEW HAMPSHIRE" OR "NEW JERSEY" OR "NEW MEXICO" OR "NEW YORK" OR "NORTH CAROLINA" OR "NORTH DAKOTA" OR OHIO OR OKLAHOMA OR OREGON OR PENNSYLVANIA OR "RHODE ISLAND" OR "SOUTH CAROLINA" OR "SOUTH DAKOTA" OR TENNESSEE OR TEXAS OR UTAH OR VERMONT OR VIRGINIA OR WASHINGTON OR "WEST VIRGINIA" OR WISCONSIN OR WYOMING OR USA OR "United States" OR u.s.a. OR American* OR GUAM OR "PUERTO RICO" OR "VIRGIN ISLANDS" OR "American Samoa" OR "Northern Mariana Islands" OR "New York City" OR "Los Angeles" OR Houston OR Dallas OR Miami OR Chicago OR Dallas OR Detroit OR Seattle OR "San Francisco" OR Sacramento OR "San Diego" OR Boston OR "Washington DC" OR “district of columbia” OR Baltimore OR Pittsburgh OR Philadelphia OR Denver OR Phoenix OR "New Orleans") NOT dollar*) OR ((Northwest* OR northeast* OR southwest* OR southeast* OR midwest* OR southern OR northern OR "new england" OR "mid atlantic" OR "Great lakes region" OR Appalachia* OR “pacific northwest”) AND (USA OR "United States" OR u.s.a.))) |
|  | **#12** | #9 OR #10 OR #11 |
|  | **#13** | Exact Subject Heading: (MH "Black Persons") OR (MH "Hispanic Americans") OR (MH "Native Americans+") OR (MH "Alaska Natives") OR (MH "Poverty") OR (MH "Minority Groups") OR (MH "Socioeconomic Factors") OR (MH "Economic Status") |
|  | **#14** | Title: (“racially marginalized” OR “marginalized population*” OR “marginalized group*” OR “disadvantaged population*” OR “disadvantaged group*” OR “vulnerable population*” OR “vulnerable group*” OR “African American*” OR black OR Latina OR Latinas OR Latino* OR latinx OR chicano* OR chicana* OR “Mexican American*” OR Hispanic* OR “Puerto Rican*” OR biracial OR “bi-racial” OR “bi racial” OR “mixed race” OR indigenous OR “Native American*” OR “American Indian*” OR “native Alaskan*” OR “Alaska native*” OR “Alaskan native*” OR “Asian American*” OR “Native Hawaiian*” OR underserved OR disparit* OR inequit* OR poverty OR poor OR “low socioeconomic status” OR “low economic status” OR “low socio economic status” OR “low SES” OR “low income” OR minority OR minorities OR “socioeconomically vulnerable” OR “women of color” OR “woman of color” OR multiethnic OR “multi ethnic” OR “diverse population*” OR “diverse group*” OR “ethnic diversity” OR “racial diversity” OR “racially diverse” OR “ethnically diverse” OR “marginalized women” OR “BIPOC women” OR “chinese women” OR “Japanese women” OR “latin American women” OR “pacific islander women” OR “cross cultural” OR crosscultural OR “Spanish speak*” OR latinoamerican*) |
| **Racial and Ethnic Groups** | **#15** | Abstract: (“racially marginalized” OR “marginalized population*” OR “marginalized group*” OR “disadvantaged population*” OR “disadvantaged group*” OR “vulnerable population*” OR “vulnerable group*” OR “African American*” OR black OR Latina OR Latinas OR Latino* OR latinx OR chicano* OR chicana* OR “Mexican American*” OR Hispanic* OR “Puerto Rican*” OR biracial OR “bi-racial” OR “bi racial” OR “mixed race” OR indigenous OR “Native American*” OR “American Indian*” OR “native Alaskan*” OR “Alaska native*” OR “Alaskan native*” OR “Asian American*” OR “Native Hawaiian*” OR underserved OR disparit* OR inequit* OR poverty OR poor OR “low socioeconomic status” OR “low economic status” OR “low socio economic status” OR “low SES” OR “low income” OR minority OR minorities OR “socioeconomically vulnerable” OR “women of color” OR “woman of color” OR multiethnic OR “multi ethnic” OR “diverse population*” OR “diverse group*” OR “ethnic diversity” OR “racial diversity” OR “racially diverse” OR “ethnically diverse” OR “marginalized women” OR “BIPOC women” OR “chinese women” OR “Japanese women” OR “latin American women” OR “pacific islander women” OR “cross cultural” OR crosscultural OR “Spanish speak*” OR latinoamerican*) |
|  | **#16** | #13 OR #14 OR #15 |
|  | **#17** | #4 AND #8 AND #12 AND #16 |
|  | **#18** | #17 AND English AND Publication Year: (2005–2021) |
|  | **#19** | Exact Subject Heading: (MH "Animals+" NOT (MH "Animals+" AND MH "Human") |
|  | **#20** | Title: (mice OR mouse OR rat OR rats OR rodent* OR murine* OR dog OR dogs OR pig OR pigs OR piglet* OR swine OR porcine* OR sheep OR animal*) |
|  | **#21** | Abstract: (mice OR mouse OR rat OR rats OR rodent* OR murine* OR dog OR dogs OR pig OR pigs OR piglet* OR swine OR porcine* OR sheep OR animal*) |
|  | **#22** | Exact Subject Heading: ((MH "Dogs") OR (MH "Rats") OR (MH "Mice") OR (MH "Rodents") OR (MH "Swine") OR (MH "Cattle") OR (MH "Animal Studies") OR (MH "Models, Biological")) |
|  | **#23** | #19 OR #20 OR #21 OR #22 |
|  | **#24** | Exact Subject Heading: (MH "News" OR MH "Retracted Publication" OR MH "Retraction of Publication OR MH "Theses and Dissertations" OR MH "Protocols") |
|  | **#25** | Title: (corrigenda OR corrigendum OR erratum OR errata OR “conference abstract” OR “conference abstracts” OR “conference proceeding” OR “conference proceedings” OR editorial OR commentary OR protocol OR protocols) |
|  | **#26** | Abstract: (corrigenda OR corrigendum OR erratum OR errata OR “conference abstract” OR “conference abstracts” OR “conference proceeding” OR “conference proceedings” OR editorial OR commentary) |
|  | **#27** | #24 OR #25 OR #26 |
|  | **#28** | #18 NOT #23 |
|  | **#29** | #28 NOT #27 |
|  | **#30** | #29 AND Publication Type: Journal Article, Meta Analysis, Meta Synthesis, Review, Systematic Review & Source Title: Academic Journals |
|  |  |  |
| **Limits Applied** |  | Language: English |
|  |  | Publication year: 2005–2021 |
|  |  | Publication type: exclude letter, editorial, comment, conference abstract, conference proceeding, case study, doctoral dissertation, proceedings |
|  |  | Species: exclude animal studies |

**Database:** PsycNet (PsycINFO & PsycARTICLES)

**Vendor:** American Psychological Association

**Date of search:** February 17, 2022

**Limits:** Language: English; Publication year: 2005–2021; Document type: exclude letter, editorial, comment, conference abstract, conference proceeding, dissertation.

**Notes:** Due to the limitations of searching PsycNet, the search strategy limiting to United States was excluded.

| **Concept** | **Search Set** | **Search Terms Used** |
| --- | --- | --- |
|  | **#1** | Index Terms: {Menopause} |
| **Menopause** | **#2** | Title: (menopause OR menopausal OR “peri-menopaus*” OR perimenopaus*) |
|  | **#3** | Abstract: (menopause OR menopausal OR “peri-menopaus*” OR perimenopaus*) |
|  | **#4** | #1 OR #2 OR #3 |
|  | **#5** | Index Terms: ({Mental Health} OR {Mental Status} OR {Mental Disorders} OR {Anxiety Disorders} OR {Bipolar Disorder} OR {Psychosis} OR {Serious Mental Illness} OR {Affective Disorders} OR {Disruptive Mood Dysregulation Disorder} OR {Premenstrual Dysphoric Disorder} OR {Schizoaffective Disorder} OR {Anxiety Disorders} OR {Obsessive Compulsive Disorder} OR {Panic Disorder} OR {Phobias} OR {Generalized Anxiety Disorder} OR {Panic Attack} OR {Cyclothymic Disorder} OR {Mania} OR {Bipolar II Disorder} OR {Bipolar I Disorder} OR {Borderline Personality Disorder} OR {Psychosis} OR {Schizophrenia} OR {Mild Cognitive Impairment} OR {Cognitive Impairment} OR {Posttraumatic Stress Disorder} OR {Suicidal Ideation} OR {Domestic Violence} OR {Child Abuse} OR {Elder Abuse} OR {Intimate Partner Violence} OR {Sexual Abuse} OR {Physical Abuse} OR {Battered Females} OR {Depression (Emotion)} OR {Major Depression} OR {Dysthymic Disorder}) |
| **Mental Health Conditions** | **#6** | Title: (“mental illness*” OR “mental disorder*” OR “mental health” OR “mentally ill” OR “mood disorder*” OR “affective disorder*” OR depression OR depressed OR “depressive disorder*” OR psychosis OR psychoses OR “psychotic disorder*” OR “schizoaffective disorder*” OR anxiety OR “panic disorder*” OR phobia* OR “phobic disorder*” OR schizophren* OR bipolar OR “manic disorder*” OR “manic depressive” OR “obsessive compulsive disorder*” OR “disruptive mood dysregulation disorder*” OR “borderline personality disorder*” OR “Dysthymic Disorder*” OR “Cyclothymic Disorder*” OR “family violence” OR “domestic violence” OR “spousal abuse” OR “spouse abuse” OR “domestic abuse” OR “elder abuse” OR “child abuse” OR “partner violence” OR “battered woman” OR “battered women” OR PTSD OR “post traumatic stress disorder*” OR “posttraumatic stress disorder*” OR “premenstrual dysphoric disorder*” OR “premenstrual dysphoric syndrome*” OR “pre-menstrual dysphoric disorder*” OR “co occurring substance use” OR “cooccurring substance use” OR “cognitive decline*” OR “cognitive dysfunction*” OR “cognitive impairment*” OR “Mild Neurocognitive Disorder*” OR “suicidal ideation*” OR “suicidal thought*” OR “suicidal behavior*” OR “suicide ideation*” OR “sexual abuse” OR “physical abuse” OR “physical violence”) |
|  | **#7** | Abstract: (“mental illness*” OR “mental disorder*” OR “mental health” OR “mentally ill” OR “mood disorder*” OR “affective disorder*” OR depression OR depressed OR “depressive disorder*” OR psychosis OR psychoses OR “psychotic disorder*” OR “schizoaffective disorder*” OR anxiety OR “panic disorder*” OR phobia* OR “phobic disorder*” OR schizophren* OR bipolar OR “manic disorder*” OR “manic depressive” OR “obsessive compulsive disorder*” OR “disruptive mood dysregulation disorder*” OR “borderline personality disorder*” OR “Dysthymic Disorder*” OR “Cyclothymic Disorder*” OR “family violence” OR “domestic violence” OR “spousal abuse” OR “spouse abuse” OR “domestic abuse” OR “elder abuse” OR “child abuse” OR “partner violence” OR “battered woman” OR “battered women” OR PTSD OR “post traumatic stress disorder*” OR “posttraumatic stress disorder*” OR “premenstrual dysphoric disorder*” OR “premenstrual dysphoric syndrome*” OR “pre-menstrual dysphoric disorder*” OR “co occurring substance use” OR “co-occurring substance use” OR “cooccurring substance use” OR “cognitive decline*” OR “cognitive dysfunction*” OR “cognitive impairment*” OR “Mild Neurocognitive Disorder*” OR “suicidal ideation*” OR “suicidal thought*” OR “suicidal behavior*” OR “suicide ideation*” OR “sexual abuse” OR “physical abuse” OR “physical violence”) |
|  | **#8** | #5 OR #6 OR #7 |
|  | **#9** | Index Terms: ({Blacks} OR {Minority Groups} OR {Latinos/Latinas} OR {Multiracial} OR {People of Color} OR {American Indians} OR {Alaska Natives} OR {Hawaii Natives} OR {Mexican Americans} OR {Marginalized Groups} OR {Poverty} OR {Disadvantaged} OR {Economic Disadvantage} OR {Economic Inequality} OR {Lower Income Level} OR {Socioeconomic Status} OR {Family Socioeconomic Level} OR {Income Level} OR {Lower Class} OR {Social Class} OR {Minority Groups}) |
| **Racial and Ethnic Groups** | **#10** | Title: (“racially marginalized” OR “marginalized population*” OR “marginalized group*” OR “disadvantaged population*” OR “disadvantaged group*” OR “vulnerable population*” OR “vulnerable group*” OR “African American*” OR black OR Latina OR Latinas OR Latino* OR latinx OR chicano* OR chicana* OR “Mexican American*” OR Hispanic* OR “Puerto Rican*” OR biracial OR “bi-racial” OR “bi racial” OR “mixed race” OR indigenous OR “Native American*” OR “American Indian*” OR “native Alaskan*” OR “Alaska native*” OR “Alaskan native*” OR “Asian American*” OR “Native Hawaiian*” OR underserved OR disparit* OR inequit* OR poverty OR poor OR “low socioeconomic status” OR “low economic status” OR “low socio economic status” OR “low SES” OR “low income” OR minority OR minorities OR “socioeconomically vulnerable” OR “women of color” OR “woman of color” OR multiethnic OR “multi ethnic” OR “diverse population*” OR “diverse group*” OR “ethnic diversity” OR “racial diversity” OR “racially diverse” OR “ethnically diverse” OR “marginalized women” OR “BIPOC women” OR “chinese women” OR “Japanese women” OR “latin American women” OR “pacific islander women” OR “cross cultural” OR crosscultural OR “Spanish speak*” OR latinoamerican*) |
|  | **#11** | Abstract: (“racially marginalized” OR “marginalized population*” OR “marginalized group*” OR “disadvantaged population*” OR “disadvantaged group*” OR “vulnerable population*” OR “vulnerable group*” OR “African American*” OR black OR Latina OR Latinas OR Latino* OR latinx OR chicano* OR chicana* OR “Mexican American*” OR Hispanic* OR “Puerto Rican*” OR biracial OR “bi-racial” OR “bi racial” OR “mixed race” OR indigenous OR “Native American*” OR “American Indian*” OR “native Alaskan*” OR “Alaska native*” OR “Alaskan native*” OR “Asian American*” OR “Native Hawaiian*” OR underserved OR disparit* OR inequit* OR poverty OR poor OR “low socioeconomic status” OR “low economic status” OR “low socio economic status” OR “low SES” OR “low income” OR minority OR minorities OR “socioeconomically vulnerable” OR “women of color” OR “woman of color” OR multiethnic OR “multi ethnic” OR “diverse population*” OR “diverse group*” OR “ethnic diversity” OR “racial diversity” OR “racially diverse” OR “ethnically diverse” OR “marginalized women” OR “BIPOC women” OR “chinese women” OR “Japanese women” OR “latin American women” OR “pacific islander women” OR “cross cultural” OR crosscultural OR “Spanish speak*” OR latinoamerican*) |
|  | **#12** | #9 OR #10 OR #11 |
|  | **#13** | #4 AND #8 AND #12 |
|  | **#14** | #13 AND Language: English AND Publication year: 2005–2021 |
|  | **#15** | #14 AND Document type: Journal Article |
|  |  |  |
| **Limits Applied** |  | Language: English |
|  |  | Publication year: 2005–2021 |
|  |  | Document type: exclude letter, editorial, comment, conference abstract, conference proceeding, dissertation. |
